# Rollover Cyclometalation as a Valuable Tool for Regioselective C–H Bond Activation and Functionalization

**DOI:** 10.3390/molecules26020328

**Published:** 2021-01-10

**Authors:** Antonio Zucca, Maria I. Pilo

**Affiliations:** Department of Chemistry and Pharmacy, University of Sassari, via Vienna 2, 07100 Sassari, Italy; mpilo@uniss.it

**Keywords:** rollover cyclometalation, C–H bond activation, C–H bond functionalization

## Abstract

Rollover cyclometalation constitutes a particular case of cyclometallation reaction. This reaction occurs when a chelated heterocyclic ligand loses its bidentate coordination mode and undergoes an internal rotation, after which a remote C–H bond is regioselectively activated, affording an uncommon cyclometalated complex, called “rollover cyclometalated complex”. The key of the process is the internal rotation of the ligand, which occurs before the C–H bond activation and releases from coordination a donor atom. The new “rollover” ligand has peculiar properties, being a ligand with multiple personalities, no more a spectator in the reactivity of the complex. The main reason of this peculiarity is the presence of an uncoordinated donor atom (the one initially involved in the chelation), able to promote a series of reactions not available for classic cyclometalated complexes. The rollover reaction is highly regioselective, because the activated C–H bond is usually in a symmetric position with respect to the donor atom which detaches from the metal stating the rollover process. Due to this novel behavior, a series of potential applications have appeared in the literature, in fields such as catalysis, organic synthesis, and advanced materials.

## 1. Introduction

Metal-mediated activation and functionalization of C–H bonds are fundamental topics in organometallic chemistry. Several efforts have been done in the past decades to shed light onto the mechanisms implied in the process. The intramolecular version of C–H bond activation, i.e., cyclometalation [1], has been extensively studied in recent decades for several reasons: the reaction is easier to study than the analogous inter-molecular counterpart, but occurs with comparable mechanisms; the products of the reactions, the so-called cyclometalated complexes, possess an additional stability due to chelated effect and have shown an ample spectrum of potential applications in different fields, from homogeneous catalysis to advanced materials and biomedicine. Worth of note, the cyclometalation reaction is usually highly regioselective, as, for example, in the case of ortho-metalation.

In cyclometalation, the C–H bond activation is usually a heteroatom-assisted process, involving classical donors such as N, O, P, S, even though also C-assisted cyclometalations are known. The initial coordination of the donor atom holds the C–H bond close to metal, facilitating its activation. As a consequence, the classical cyclometalation pathway generally consists of two consecutive steps: (i) initial coordination of a donor atom and (ii) subsequent intramolecular activation of a C–H bond to form a metallacycle [2]. Closely related to cyclometalation is the directed ortho-metalation [3], where regioselective ortho C–H bond activation is heteroatom-assisted; in this case, however, the metallacycle is not preserved due to the nature of the heteroatom-metal bond.

The first cyclometalation reaction appeared in 1955, described as an “aluminum organometallic inner complex” [4], but the term “cyclometalation” was coined only in 1973 by Trofimenko [5].

The properties of cyclometalated complexes may be tuned in a number of ways. In these species, the correlation between ligands and complexes properties can be remarkably high, so that modification of the stereoelectronic parameters of the cyclometalated ligand usually allows a refined tuning of the overall properties of the complex.

In addition to classical cyclometalated complexes, a series of variations on the basic theme lead to several subclasses, such as that of the so-called “pincer” complexes [6], where the metallacycle is comprised in a tridentate system (e.g., N^C^N or C^N^C). A less-common special class of cyclometalated species, the so-called “rollover” complexes [7], has assumed a particular interest in recent years, having peculiar properties that distinguish it from the other cases.

Consequently, the reaction which leads to these compounds, i.e., rollover cyclometalation, has attracted attention. In rollover metalation, a chelated ligand, usually a heteroaromatic bidentate donor such as 2,2′-bipyridine, may choose to coordinate in the classical way, i.e., as a chelated ligand, or, after internal rotation and deprotonation, as an N^C anionic cyclometalated ligand. The key of the process is the internal rotation of the ligand, which allows activation of a C–H bond initially distant from the metal.

**Figure 1:**
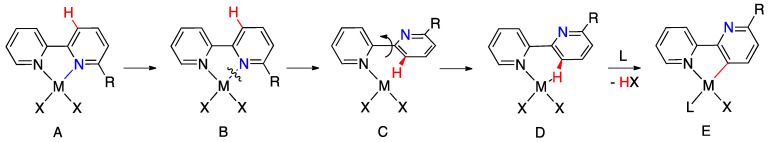
Rollover cyclometalation: fundamental steps.

Taking 2,2′-bipyridine as a model, roll-over cyclometalation follows a series of elementary steps (Figure 1): First, the ligand will behave as a classical N^N chelate (A), then one of the nitrogen donors decoordinates (B), allowing rotation along the C–C bond which connects the two heteroaromatic rings (C). The internal rotation allows activation of the initially remote C(3)-H bond (D), to afford the final cyclometalated product (E). The reaction is highly regioselective, because it allows activation of a C–H bond on the other side with respect to the detached nitrogen, usually, as in this case, in a symmetric position.

It is important, for a “true” rollover cyclometalation, to start from a chelated complex from which C–H bond activation occurs in a succeeding coordinatively unsaturated complex. In their review of 201 [7], Butschke and Schwarz coined the term “pseudo-rollover” cyclometalation, for those cases that proceed in a mechanistically different mode, for instance, starting from a monodentate complex, without chelation; in this case, a simple cyclometalation occurs, but the final complex may be also classified as a rollover species. In other cases of pseudo-rollover cyclometalation, the ligand has been previously activated, e.g., by lithiation or other methods.

Several studies have been made in order to elucidate the mechanism of the rollover process. The final step, i.e., metal-mediated C–H activation, can follow different mechanisms depending on the metal involved and the rollover ligand implied. This aspect will be treated in Section 3.

Even though 2,2′-bipyridines have initially been the most studied ligands in this field, the reaction is not restricted to these family of compounds and interests a vast series of heteroaromatic compounds. As a general rule, every bidentate heterocyclic ligand having a C–H bond in a symmetric position to one of the donor atoms, flexible enough to allow internal rotation, can potentially give rollover cyclometalation. Ligands without this symmetry are also able to follow a rollover pathway, provided the presence of an available C–H bond “on the other side”.

**Figure 2:**
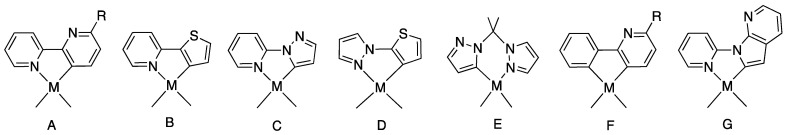
Selection of rollover complexes.

In Figure 2, we report a selection of rollover complexes derived from 2,2′-bipyridines (A), 2-(2-thienyl)pyridine (B), 2-(1-pyrazolyl)pyridine (C), 1-(2-thienyl)-1*H*-pyrazole (D), pyrazolylmethanes (E), 2-phenyl-pyridine (F). The last example, G, derived from *N*-(2′-pyridyl)-7-azaindole, is a case of a rollover complex without the above-mentioned N/C–H symmetry.

Having defined reaction and ligands, some considerations follow, considering a generic E, E chelating ligand (E = N, S, O, etc.):

- roll-over cyclometalation is favored by the presence of one weak M–E bond in the starting chelated complex. The weakness may be related to steric or electronic factors: as a consequence, the reaction will be favored by bulky substituents in adjacent position to E, electron-withdrawing groups in the ring and ligands with strong *trans*-influence coordinated in *trans* to E. *Trans*-influence and *trans*-effect may have dramatic effect on the behavior of some ligands and in catalytic applications;
- formation of a strong M–C bond will thermodynamically favor the rollover process toward classic chelation. For this reason, late and heavy transition metals such as iridium and platinum are favored;
- a second peculiarity of rollover cyclometalation is found in the mechanism of the cyclometalation reaction: in the step from chelate to monodentate coordination, one vacant coordination site is formed. This free site for coordination lacks in the classical cyclometalation reaction;
- the final rollover product has a free heteroatom (N in the majority of cases) able to undergo successive reactions or interactions: coordination, protonation, hydrogen-bond interactions, return to initial chelation, etc. Here lies the crucial difference between rollover and classical cyclometalated complexes: three consequences of this aspect are:
  - the retro-rollover process, i.e., the reverse reaction of rollover cyclometalation (see Section 4.1.5): In these steps, one hydrogen is lost and regained, with interesting applications in catalytic hydrogen transfer reactions (see Section 6.2).
  - The double rollover cyclometalation, which allows for the synthesis of planar, highly delocalized bi- or polynuclear complexes (see Section 4.1.3).
  - The protonation of the free donor usually allows the formation of uncommon NHC (nitrogen heterocyclic carbenes), formally neutral ligands, which are isomers of the neutral starting ligand. This option enters rollover complexes into the family of “ligands with multiple personalities” [8] rather than being mere spectator ligands (see Section 4.1.5).
- Finally, it should be noted that “rollover” ligands are deprotonated forms of the starting neutral ligands, whose chelated complexes are usually stable and difficult to activate. The strength of the M–E and M–C bonds, as well as mechanisms of C–H bond activation vary from metal to metal, so that each metal will have a different story. This accounts for the difficulties to find general rules for rollover cyclometalation.

For all these reasons, rollover cyclometalation constitute a potent instrument for the regioselective C–H bond activation and functionalization of the C–H bond located in positions usually not available for metal-mediated C–H activation, as well as for the synthesis of compounds with novel properties. This review will be focused on the application of rollover metalation to regioselective C–H bond activation and functionalization processes. Both stochiometric and catalytic activation/functionalization will be treated. However, in order to furnish a more ample vision of the reaction, we will also report examples of reactivity and applications of rollover cyclometalated complexes.

## 2. History of Rollover Cyclometalation

**Figure 3:**
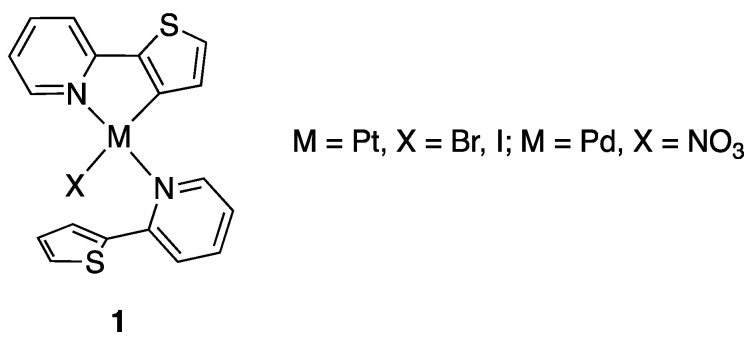
The first rollover complexes.

To the best of our knowledge, the first rollover complex appeared in the literature in 1975, only two years after Trofimenko’s recognition of the cyclometalation reaction: Giordano and Rasmussen simply described the Pt(II) and Pd(II) rollover-cyclometalated products of 2-(2′-thienyl)pyridine, **1** (Figure 3), as “Compounds Containing Metal-Carbon Bonds” [9] without recognizing their novelty.

**Figure 4:**
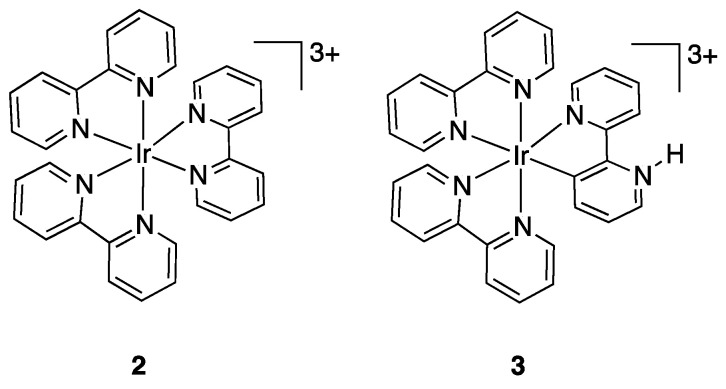
The first iridium rollover complex.

Soon later, in 1977, Watts and coworkers reported the synthesis of an Ir(III) species, initially described as an iridium(III) complex containing a monodentate 2,2′-bipyridine, related to the well-known cationic complex [Ir(bpy)_3_]^3+^ (complex **2**) [10]. Only after a long and controversial debate, several years later, the nature of the complex was determined by means of X-ray and NMR studies [11,12,13,14,15] as having two N, N chelated bipyridines and one N, C cyclometalated bipy, the latter being a neutral ligand, due to protonation of the uncoordinated nitrogen (Figure 4, complex **3**).

**Figure 5:**
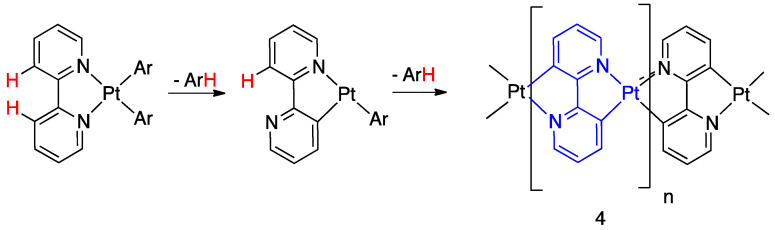
Thermal rearrangement of [Pt(bipy)(Ar)_2_] affording the organometallic polymer 4. Adapted with permission from *J. Chem. Soc. Chem. Commun*. **1985**, 609–611. Copyright (1985) Royal Society of Chemistry.

The term roll-over was coined by Skapski, Sutcliffe, and Young in 1985 [16], describing the thermal rearrangement of (Ar)_2_(bpy)platinum(II) complexes: no mononuclear species were isolated but the metalation was confirmed by the X-ray structure of a dinuclear species. Interestingly, the reaction finally leads to the isolation of the largely delocalized organometallic polymer [Pt(bipy-2*H*)]_n_, **4**, having bridging double-rollover bipyridines (Figure 5). Kinetic measurements showed that the rollover reaction is faster starting from [Pt(bipy)(4-*t*-but-Ph)_2_] in comparison to [Pt(bipy)(4-CF_3_-Ph)_2_].

Since then, the term roll-over was not used until 1999, when Minghetti’s group reported a series of Pt(II) and Pd(II) rollover complexes with substituted 2,2′-bipyridines (see later) [17].

From then, an increasingly number of papers have appeared in the literature, demonstrating, inter-alia, by the publication of two reviews completely dedicated to the subject [7,18] with other reviews also dealing in part with the same theme [19]. Potential applications in organic synthesis, homogeneous catalysis, biomedicine, and advanced materials are revealing the hidden potentialities in this field.

## 3. Mechanism of Rollover Cyclometalation

The activation of C–H bonds in homogeneous systems has been a matter of study for many years. From a mechanistic point of view, it is generally accepted that, in cyclometalation reactions, C–H bond activation follows three major pathways: electrophilic activation (or heterolytic cleavage), oxidative addition, and σ-bond metathesis [1,2,20].

The operating mechanism depends on several factors, such as the electronic nature of the L_n_M fragment and the nature of the C–H bond, and in most cases, it is far from being determined. The comprehension of the mechanism is complicated by the fact that subtle structural modifications in the ligand or in the metal center may deeply influence the reaction pathway.

C–H bond activation via electrophilic activation is common for electron-poor late transition metals such as palladium(II), and, to some extent, also platinum(II). This pathway is operative especially in the case of C(sp^2^)–H activations in aromatic rings. Mechanistic studies on aromatic C–H bond activation revealed that electron-donating substituents on the ring favors the metal-mediated activation, in analogy to electrophilic aromatic substitutions in organic compounds.

In contrast, oxidative addition pathways require electron-rich low-valent metal centers and is common for late transition metals, such as Ru, Os, Rh, Ir, Pt; also, it seems to be preferred in C(alkyl)–H bond activations.

The σ-bond metathesis pathway has been considered a prevalent mechanism in the case of electron-poor metal centers such as high-valent early transition metals. In the case of late transition metals, such as Pt(II) and Pd(II), the metal supports the stabilization of the σ complex and the process has been designated σ-CAM, σ-complex assisted metathesis.

It is worth to note that an oxidative addition pathway produces a hydride complex; however, subsequent reductive eliminations of HX (X = coordinated anionic ligand) may afford final cyclometalation products without the expected coordinated hydride, making it difficult to distinguish it from the other two mechanisms.

A final, particular case regards trasmetalation reactions (in this case, transcyclometalation) which do not involve a direct C–H bond activation but require a starting organometallic compound for the exchange reaction M-C + M’-X → M-X + M’-C.

In rollover cyclometalation, some aspects of the process are different from classical cyclometalation. Most the experimental and DFT studies on the operating mechanism regarded platinum(II) bipyridine complexes. Changing metal and bidentate ligand may obviously result in a different mechanism. Starting from the usual N^N adduct, detachment of the nitrogen and internal rotation are the crucial differences between classical and rollover cyclometalation; in the latter, a coordination position is liberated by the nitrogen, differently from the classical case. These two steps allow interaction of the C(3)-H bond with the metal center.

As for rollover cyclometalation, the operative mechanisms of the C–H bond activation step are expected to be the same as in classical cyclometalation. All three mechanisms have been postulated to be effective with different metals and ligands.

**Figure 6:**
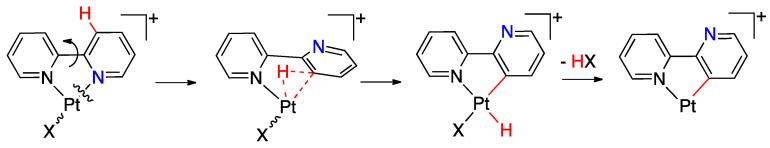
Proposed mechanism for the gas-phase rollover cyclometalation of [Pt(bipy)X]+.

In the case of electron-rich Pt(II) complexes, such as [PtMe_2_(DMSO)_2_] or [PtMe_2_(SMe_2_)]_2_, an oxidative addition pathway has been proposed by several authors. Schwarz and coworkers conducted a mechanistic study, based on CID-MS (collision induced dissociation mass spectrometry) and DFT calculations, on the gas-phase rollover transformation of [M(bipy)X]^+^ to [M(bipy-*H*)]^+^ species (M = Ni, Pd, Pt; X = CH_3_, Cl), founding a clear preference for the oxidative-addition/reductive-elimination pathway for Pt (Figure 6), whereas in the case of Ni(II), an s-bond metathesis is preferred. For palladium, the preferred mechanism depends on the nature of the anionic ligands X: s-bond metathesis is favored for [Pd(bipy)Me]^+^, whereas oxidative addition/reductive elimination is slightly preferred in the case of [Pd(bipy)(Cl)]^+^ [21].

The studies in the solution essentially regarded [PtMe_2_(DMSO)_2_] and [PtPh_2_(DMSO)_2_] derivatives. Young and coworkers, in their pivotal work in 1985 [16], found that electron richer Pt(II) derivatives [PtAr_2_(DMSO)_2_] reacted faster than electron poorer analogues (Ar = 4-^t^Bu-C_6_H_4_; 4-CF_3_-C_6_H_4_).

Minghetti, Zucca, and co-workers working with 2,2′-bipyridine and several 5- and 6-substituted 2,2′-bipyridines found via NMR spectroscopy that the process follows a consecutive reaction mechanism, which begins with initial N^N chelation to produce [Pt(N^N)(CH_3_)_2_], followed by rapid methane liberation [17,22]. For the final steps, i.e., the crucial C–H bond activation and successive elimination of methane, the proposed mechanism involves, also in the solution, an oxidative-addition/reductive-elimination sequence which eventually liberates methane. No sign of hydride intermediates was observed in the solution in the case of platinum, however, Zuber and Pruchnik studied the behavior in the solution of the [Rh(bipy)_2_Cl] complex reporting the NMR detection of rhodium-hydride intermediates in a reversible rollover/retro-rollover process (see Section 4.1.7). Analogously, the reaction of a Rh(I) NHC complex, [Rh(NHC)(C_2_H_4_)(Cl]_2_ with 2,2′-biquinoline gave, at room temperature, the rollover hydride [Rh(NHC)(Cl)(H)(N^C)], where N^C is a rollover coordinated 2,2′-biquinoline [23].

The experimental data indicate that, in the case of the [Pt(N^N)Me_2_] intermediate, de-coordination of nitrogen is favored by the high *trans*-influence of the methyl ligand, a bulky and/or electron attracting substituent in position 6 (which increases the steric congestion in the square-planar and/or reduces the donor ability of the nitrogen). In particular, the CF_3_ substituent favors the reaction also in 5-position, furnishing a clear electronic effect on the outcome of the reaction.

Oxidative-addition mechanism was also proposed by Wang and coworkers in the rollover activation of another “PtMe_2_” adduct [Pt(N^N)Me_2_] (See Section 4.2.1, complex **76**) [24]. The authors assumed an oxidative-addition reductive-elimination pathway for the process, based on the electron-richness of the Pt center in the three-coordinate intermediate and on the formation of only one isomer as a result of the process. An oxidative-addition pathway was also proposed for the Rh-catalyzed selective functionalization of 2-(2-thienyl)pyridine (see Section 6.2.1) [25].

At variance, Thiel and coworkers, in their review on the argument [18], proposed that the final step of the roll-over cyclometalation of aromatic nitrogen donors is analogous to a classical organic S_E_Ar reaction, where the metal acts as the electrophile. Therefore, “the more electron-rich the ring system is, the more reactive it should be.“ This is not in line with the extreme rapidity of the reaction of 6-CF_3_-2,2′-bipyridine with [PtMe_2_(DMSO)_2_] (and even 5-CF_3_-2,2′-bipyridine) but it should be suitable on the basis that different metals (and also different metal precursors of the same metal) may act following different reaction mechanisms. In agreement with an electrophilic mechanism is the rollover cyclometalation of 2,2′:6′,2″-terpyridine promoted by Pt(II), where a two-fold C–H bond activation occurs on the central pyridine ring. After the first cyclometalation, the central pyridine (with a higher electron density due to metalation) is activated towards a second metalation, which occurs faster than the first one [26]. Accordingly, reaction of [Ir(Cp*)Cl_2_]_2_ with 2-(2-dimethylaminopyrimidin-4-yl)pyridine gave the rollover cationic complex [Ir(Cp*)N^C)Cl]^+^, a behavior in agreement with an electrophilic aromatic substitution [27].

On the other hand, a σ-bond metathesis pathway is suggested by a DFT analysis of the room-temperature rollover cyclometalation of palladium(II) complexes with phosphine pyrazole pincer ligands P^C^N (PCN = 1-[3-[(di-*tert*-butylphosphino) methyl]phenyl]-1*H*-pyrazole) and = 1-[3-[(di-*tert*-butylphosphino)methyl]phenyl]-5-methyl-1*H*-pyrazole, see Section 4.2.1) [28]. DFT data revealed low energy barriers for C–H activation through a σ-bond metathesis reaction, in line with the experimental outcomes.

Finally, quite recently, a C–H bond rollover activation which precedes nitrogen coordination was proposed for the reactions of 2,2′-bipyridines with a hexahydride bipyridine osmium complex (see Section 4.1.7, Osmium). The C–H bond activation step consists in a hydride-mediated heterolytic cleavage of the C–H bond, promoted by the electrophilic Os(IV) complex formed by H_2_ reductive elimination. C–H bond selectivity is the consequence of nitrogen trapping of the intermediate formed by C–H activation. Comparable considerations are likely true for the corresponding reaction of a pentahydride iridium complex [29].

## 4. C–H Bond Activation Through Rollover Cyclometalation

During the last years, several metals showed the ability to promote rollover cyclometalation with an ample series of substrates. A variety of rollover cyclometalated complexes have been synthesized and studied. In this chapter, we will examine stoichiometric cyclometalation reactions, dividing the discussion according to typologies of ligands and metal involved.

### 4.1. Bipyridine Complexes

In 2020, 2,2′-bipyridine (bpy) celebrated 132 years from its discovery, which occurred in 1888. Bipyridine is undoubtedly one of most important ligands in coordination chemistry, so that some years ago, a review recognized it as “the most widely used ligand” [30]. It is well-known that the common coordinative behavior of 2,2′-bipyirine is as a chelated N^N ligand, even though monodentate and bridging complexation is recognized, although rare.

For these reasons, the new examples of metalating behavior aroused interest, and researchers struggled to find methods to induce this ligand to leave the comfortable N^N behavior for the “new way”. Although several ligands can behave in a rollover mode, as seen before, 2,2′-bipyridine remains the prototypical and most important ligand in this field. It is also worth to note that an N^C-rollover coordinated bipyridine is not precisely (not only, at least) a bipyridine coordinated in a new way, but is a formally anionic C(3)-deprotonated 2,2′-bipyridyl.

#### 4.1.1. Platinum and Palladium Complexes

After Young’s pivotal paper, the term “rollover”, along with the concept itself of “rollover cyclometalation” was not recognized for several years, until in 1999 Minghetti’s group in Sassari re-submitted the term, reporting the rollover cyclometalation of 6-substituted-2,2′-bipyridines (6-R-2,2′-bipyiridines, R = isopropyl, neopentyl, and *tert*-butyl) by means of Pt(II) and Pd(II) precursors [17].

**Figure 7:**
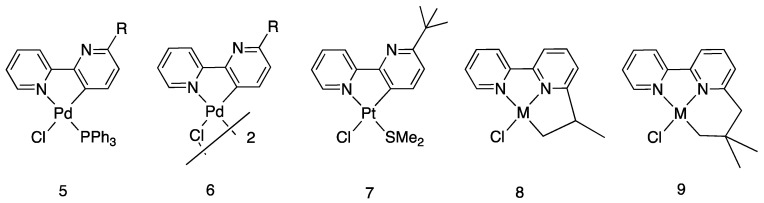
Rollover and tridentate N^N^C complexes with 6-substituted 2,2′-bipyridines.

The paper reported the first case of bipyridine rollover cyclometalation promoted by palladium: starting from Pd(II) acetate, a well-known cyclometalating precursor, mono and dinuclear complexes were isolated and characterized (**5** and **6**, R = isopropyl and neopentyl, Figure 7). The substituent in position 6 resulted in being crucial for the reaction, likely for steric reasons. In the same paper, following Young’s discovery, *trans*-[PtClMe(SMe_2_)_2_] was reacted with 6-*tert-*butyl-2,2′-bipyridine affording the rollover complex **7**. In addition, in this case, under the reaction conditions studied, the bulky alkyl substituent in position 6 resulted in being crucial for the outcome of the reaction.

It is worth to note that under different reaction conditions, e.g., from [MCl_4_]^2−^ precursors in water/HCl (M = Pd, Pt), the reaction proceeded in a different way, activating a C–H bond in the substituent to give the corresponding [M(N^N^C)Cl] tridentate complexes **8** and **9** (M = Pd, Pt) [31,32,33,34,35]. The topic of C–H regioselectivity in substituted bipyridines and related ligands is complex, being the reaction outcome driven by several factors, and will be treated later.

**Figure 8:**
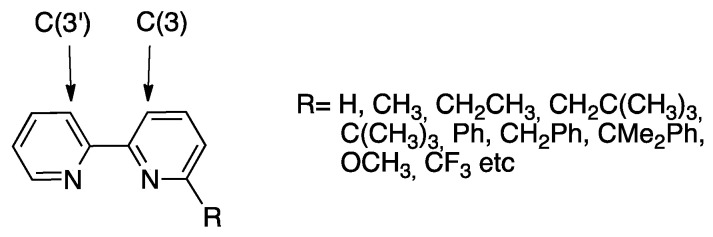
6-substituted-2,2′-bipyridines. C(3) and C(3′) positions, involved in rollover activation, are shown.

After a series of studies dedicated to related polycyclic bipyridine systems, such as 2,2′:6′,2″-terpyridine and 6,6′-Ph_2_-bipyridine (see later), the investigation was extended to a series of 6-substitued bipyridines (bpy^R^, Figure 8) [22] and compared to unsubstituted 2,2′-bipyridine [36], evincing the role of the substituent in the reaction.

**Figure 9:**
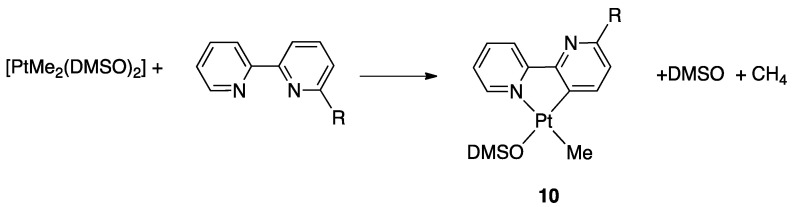
Rollover cyclometalation of 6-substituted 2,2′-bipyridines with [PtMe_2_(DMSO)_2_]. Adapted with permission from *Organometallics* **2003**, 22, 23, 4770–4777. Copyright (2003) American Chemical Society.

In the Sassari Laboratory of Organometallic Chemistry, the study involved the reaction a series of Pt(II) precursors such as [PtMe_2_(DMSO)_2_], [PtPh_2_(DMSO)_2_], [PtMeCl(DMSO)_2_], [PtCl_2_(DMSO)_2_] with several substituted bipyridines. The most electron-rich complex, i.e., [PtMe_2_(DMSO)_2_], showed to be the best Pt(II) precursor for the synthesis of bipyridine rollover complexes **10** (Figure 9). A clear steric influence of the substituent was evidenced by the fast reaction of [PtMe_2_(DMSO)_2_] with 6-*tert*-butyl-2,2,′-bipyridine, with C(3)–H bond rollover activation even at room temperature. In comparison, 2,2′-bipyridines with less steric-demanding substituents such as Me or neo-pentyl, needed higher temperatures (acetone, 40–60 °C). In the absence of a substituent, i.e., with 2,2′-bipyridine, the activation occurs only under hasher conditions, such as refluxing toluene [36]. A kinetic study showed that the rollover process entails a consecutive reaction through the detectable intermediate Pt(bpy^R^)Me_2_.

According to the Pt/bipy molar ratio, the reaction afforded the mononuclear compound (1:1 Pt:bipy molar ratio) or the dinuclear “double rollover” complex (2:1 Pt:bipy molar ratio) by means of double C(3)-H and C(3′)-H bond activation. In the dinuclear complex, a twofold deprotonated 2,2′-bipyridine acts as a planar, formally dianionic, delocalized ligand, connecting two metal centers (see Section 4.1.4).

**Figure 10:**
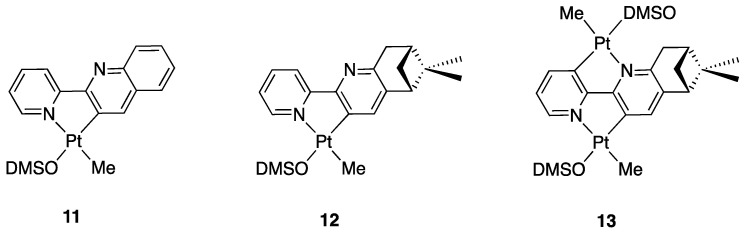
Pt(II) complexes **11**–**13**.

The succeeding studies were extended to two ligands with 6-membered fused rings: the chiral pinene-derived ligand (5S,7S)-5,7-methane-6,6-dimethyl-2-(pyridin-2-yl)-5,6,7,8-tetrahydroquinoline and the delocalized 2-(2′-pyridyl)quinoline. Both the ligands react with [PtR_2_(DMSO)_2_] (R = Me, Ph) in the same way as 6-substituted 2,2′-bipyridines: 2-(2′-pyridyl)quinoline gave the mononuclear rollover complex **11** [37], whereas the chiral pinene-derived ligand (5S,7S)-5,7-methane-6,6-dimethyl-2-(pyridin-2-yl)-5,6,7,8-tetrahydroquinoline showed to be able to give both mono- and dinuclear rollover complexes **12** and **13** [38] (Figure 10).

**Figure 11:**
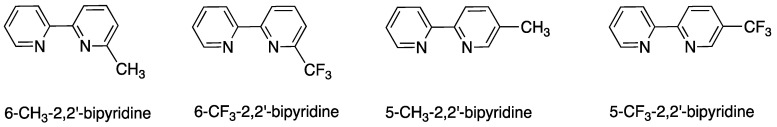
6- and 5-substituted 2,2′-bipyridines with CH_3_ and CF_3_ groups.

In order to shed light into the delicate balance between steric and electronic effects, Zucca and coworkers investigated the influence of substituents CH_3_, CF_3_, CH_2_CH_3_, and OCH_3_, [39,40,41], two couples of substituents having similar steric hindrance (CH_3_ and CF_3_; CH_2_CH_3_ and OCH_3_), but different electronic effects. For the first couple, the location of CH_3_ and CF_3_ in position 6 or 5 allows to separate, in part, electronic and steric influences (Figure 11).

The ligands were compared to unsubstituted 2,2′-bipyridine in the reaction with the electron-rich precursor [Pt(Me)_2_(DMSO)_2_]. When the substituent was located in position 6, regiospecific activation of the substituted ring occurred affording complexes **14** (R = CH_3_, CF_3_), and no sign of activation on the unsubstituted ring was observed. The electron withdrawing CF_3_ substituent in 6-CF_3_-2,2′-bipyiridine induced a noteworthy acceleration of the rollover reaction, likely due to a sum of electronic and steric factors.

**Figure 12:**
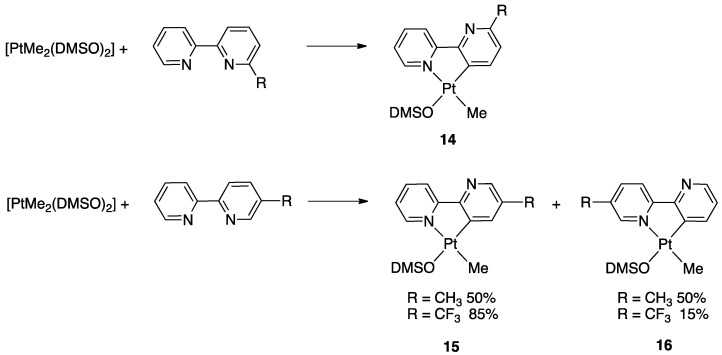
Influence of substituents in position 5 and 6 in 2,2′-bipyridines. Adapted with permission from *Organometallics* **2015**, 34, 5, 817–828. Copyright (2015) American Chemical Society.

At variance, with 5-CF_3_-2,2′-bipyiridine, the reaction outcome was independent from the steric factor related to the substituent and was not regiospecific. Two isomers were obtained due to activation of both the pyridine rings (**15** and **16**), however, with the electron-withdrawing CF_3_ group, a preference for C(3)-H activation in the substituted ring was observed (compound **15**), with a 5:1 molar ratio (Figure 12) both in refluxing acetone and toluene. Operating with 5-CH_3_-2,2′-bipyiridine, a 1:1 molar ratio between the isomers was observed.

These data indicate that an electron-poor pyridine ring is more reactive in the rollover process; together with Skapski, Sutcliffe, and Young’s observation that electron-rich Pt(II) precursors [Pt(Ar)_2_(DMSO)_2_] accelerate the rollover reaction [16], an electrophilic activation pathway should be ruled out for these reactions. Under these conditions, the CH_3_ substituent showed to have no electronic influence on the outcome of the reaction.

**Figure 13:**
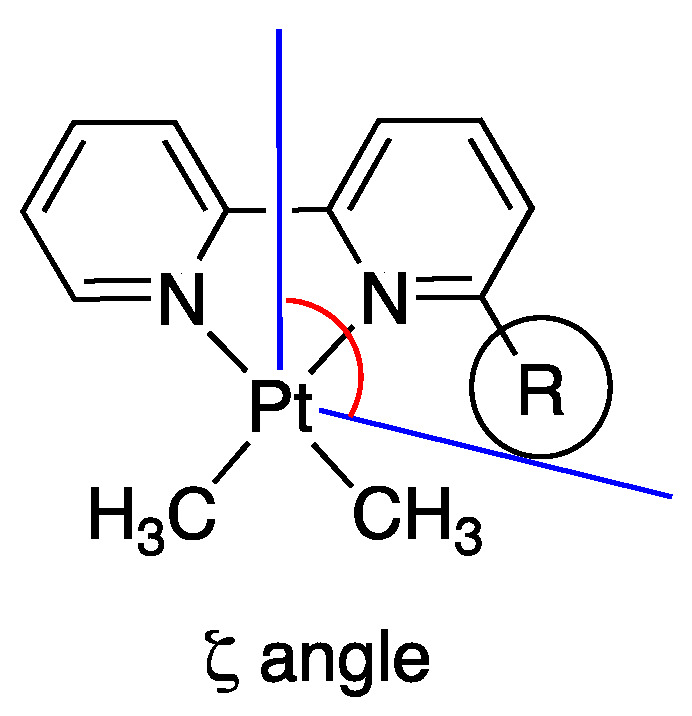
ζ angle for Pt(6-R-bipy)Me_2_ complexes.

The authors also investigated the influence of electronic factors by analyzing proton affinity data of substituted pyridines, and introduced the angle ζ in [Pt(N,N)Me_2_] adducts (Figure 13) in order to have quantitative measurement of the steric requirement on the plane of the substituted bipyridine in the intermediate N^N adducts. Unsubstituted 2,2′-bipyridine occupies 8.8° more than the theoretical 90° of a square planar coordination, having a z angle of 98.8°, a methyl in 6-Me-2,2′-bipyridine increases the value to 125.1°, whereas 6-CF_3_-2,2′-bipyridine has a value of 137.6°.

On the whole, taking account of the irreversible nature of the reaction (due to methane release), it can be deduced that a substituent in position 6, due to destabilization of the adduct, accelerates the rollover reaction, yielding regioselective C(3)–H bond activation. A CF_3_ substituent in position 6 favors the reaction for three reasons: the sterically destabilization of the N^N adduct, the electronically destabilization of the adduct (the nitrogen close to the substituent is a very poor donor), the activation of the electron poor C(3)–H bond.

The comparison of 6-methoxy-2,2′-bipyridine with 6-ethyl-2,2′-bipyridine evidenced the non-trivial influence of the methoxy substituent, having opposite inductive and mesomeric effects, both on Pt(II)-mediated rollover activation and on the properties of resulting rollover complexes [37,38].

**Figure 14:**
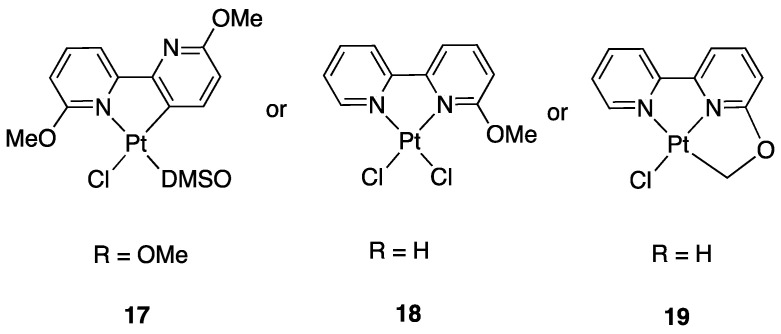
Pt(II) complexes with 6-OMe- and 6,6′-(OMe)_2_-2,2′-bipyridine.

In all the cases studied with bipyridine derivatives, it was found that the most active Pt(II) precursor is the electron-rich [PtMe_2_(DMSO)_2_] complex. The analogue phenyl derivative [PtPh_2_(DMSO)_2_] showed to be slightly less active. In contrast, the electron-poor [PtCl_2_(DMSO)_2_] does not activate the C(3)–H bond with the only exception of the di-substituted 6,6′-(OMe)_2_-2,2′-bipyridine. In this case, reaction with [PtCl_2_(DMSO)_2_] afforded the rollover complex **17** [42], whereas under the same conditions, the electron-poor [PtCl_2_(DMSO)_2_] did not activate the C(3)–H bond of the mono-substituted 6-OMe-2,2′-bipyridine affording the adduct [PtCl_2_(bpy^6OMe^)], **18**. Under harsher conditions, activation of a C–H bond in the substituent gives the classical tridentate cyclometalated complex **19** (Figure 14) [40].

**Figure 15:**
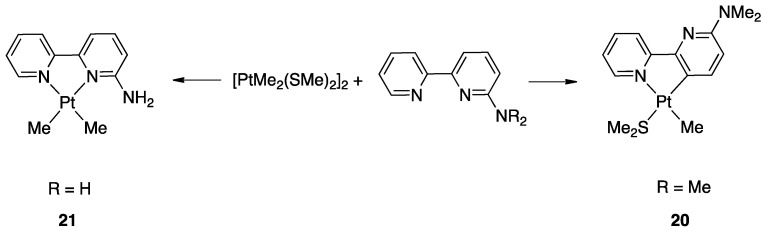
Behavior of 6-NH_2_- and 6-NMe_2_-2,2′-bipyiridine.

The influence of steric factors in rollover metalation was also observed in the reaction of 6-NH_2_-2,2′-bipyridine and 6-NMe_2_-2,2′-bipyridine with [PtMe_2_(SMe_2_)]_2_ at room temperature. The steric difference due to the methyl groups in the dimethylamino substituent drives the reaction towards the rollover cyclometalated complex **20** instead of the classical N^N bidentate coordination **21** (Figure 15) [43].

##### Regioselectivity

Regioselectivity in metal-mediated C–H bond activation is not a trivial topic; with regard to cyclometalation reactions, competition between different coordination modes (e.g., five- vs. six-membered rings, bidentate vs. tridentate coordination, M-C(sp^2^) vs. M-C(sp^3^), etc.) may occur.

**Figure 16:**
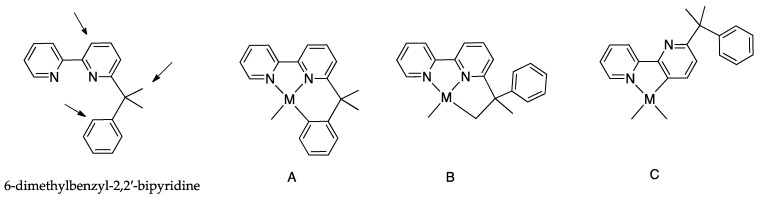
Positions of C–H bond activation in 6-dimethylbenzyl-2,2′-bipyridine and resulting Pt and Pd complexes.

The driving force of the cyclometalation reaction is often not fully understood, and subtle differences in the structure of the ligand or in reaction conditions may frequently drive the reaction towards unexpected results. As an example, Pd(II) and Pt(II) complexes are able to activate several C–H bonds in substituted 2,2′-bipyridines. In the case of 6-dimethylbenzyl-2,2′-bipyridine (Figure 16), three different positions can be activated, affording an N^N^C(sp^2^) tridentate complex with [5,6] fused rings by C(sp^2^)–H bond activation, an N^N^C(sp^3^) tridentate complex with [5] fused rings by C(sp^3^)–H bond activation, and, thirdly, an N^C cyclometalated rollover complex by C(sp^2^)–H bond activation on the pyridine C(3) atom (Figure 16 species A, B, and C, respectively) [31,33,44]. Other 6-Alkyl- and 6-benzyl- 2,2′-bipyridines show a similar behavior [33,45].

In the case of platinum(II), an important point, apart from the bulkiness of the substituent, is the electron density on the metal: electron-rich [PtMe_2_(DMSO)_2_] and [PtMe_2_(SMe_2_)]_2_ complexes showed to be the best starting material. The C–H bond activation step may follow different reaction paths (see Section 3) and in the case of methyl-platinum(II) complexes, it is expected to proceed through an oxidative addition/reductive elimination sequence [22]. In the solution, the reductive elimination step involves elimination of methane, making the process irreversible. When electron poor starting Pt(II) complex are used, such as [PtCl_4_]^2−^, classical cyclometalation occurs, likely following a different mechanism, and tridentate N^N^C complexes are formed [35].

**Figure 17:**
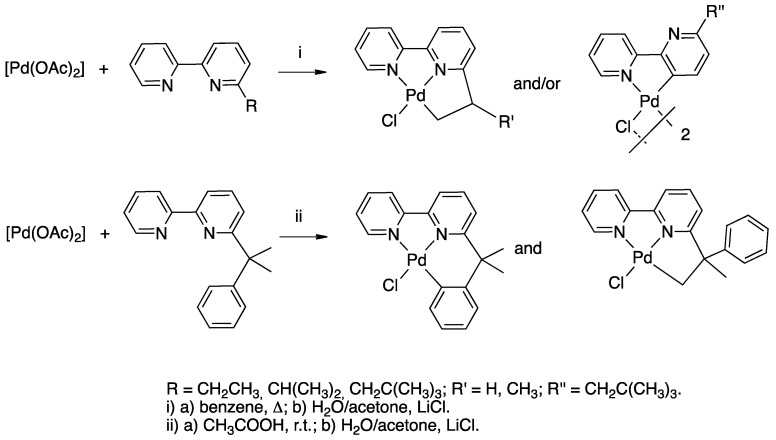
Cyclopalladation reactions with 6-substituted 2,2′-bipyridines. Adapted with permission from *Organometallics* **2000**, *19*, 21, 4295–4304. Copyright (2000) American Chemical Society.

In the case of palladium, the situation is less clear: starting from Pd acetate, the rollover reaction is not regiospecific and the N^C rollover is usually formed in competition with classic N^N^C cyclometalated complexes (Figure 17) [33]. It should be noted that, in this case, acetic acid is formed as a byproduct and the reaction is expected to be reversible, as in the case of [MCl_4_]^2−^ derivative (M = Pd, Pt; HCl as byproduct).

All the platinum-mediated cases reported so far regarded rollover reactions promoted by Pt(II) derivatives. A rare Pt(IV) rollover cyclometalation was reported by Safari and coworkers with 6,6′-dimethyl-2,2′-bipyridine. The metalation occurs under mild conditions (60 °C) starting from the electron poor Pt(IV) complex H_2_PtCl_6_, with the salt [(bipyH)_2_][PtCl_6_] as intermediate, to finally give complex [Pt(N^C)Cl_3_(DMF)] **22** (N^C = rollover-cyclometalated 6,6′-dimethyl-2,2′-bipyridine, DMF = dimethylformamide) [46]. Worthy to note, complex **22** displayed in vitro cytotoxicity, showing a higher activity than cisplatin against the colon cancer cell line, with less toxicity on normal cells.

#### 4.1.2. *N*-Functionalized Bipyridines

*N*-functionalized 2,2′- bipyridines form a special case of bipyridine derivatives, being unable to form the N^N chelated complex which lies at the origin of the rollover pathway.

**Figure 18:**
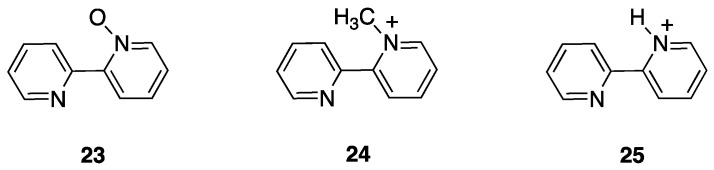
*N*-functionalized 2,2′-bipyridines.

Three cases are of interest: 2,2′-bipyridine *N*-oxide, **23**, and the *N*-methyl-2,2′-bipyridylium and *N*-protonated 2,2′-bipyridylium cations **24** and **25** (Figure 18).

**Figure 19:**
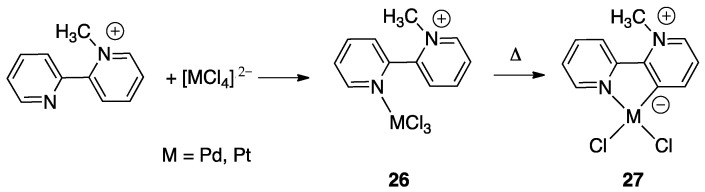
Pseudo rollover cyclometalation of the *N*-methyl-2,2′-bipyridylium cation. Reprinted (redrawn) by permission from Springer Nature, *Transition Met. Chem*. **1985**, *240*, 238–240. Substitution of [M(bpyMe)C13] and [M(bpyMe-*H*)C12] (M = Pd or Pt; bpyMe = *N*-methyl-2,2′-bipyridylium ion) with *N*-heterocycles. Wimmer, F.L.; Wimmer, S.; Delhi, N.; Facility, S.I.; Griffith, P.; Ten, R.W.M.; Langhout, J.P.; Gowda, N.M.N.; Gowda, M.N.; Naikar, S.B. COPYRIGHT 1985.

Reaction of [PdCl_4_]^2−^ and [PtCl_4_]^2−^ with the *N*-methyl-2,2′-bipyridylium ion **24** initially gave the monodentate complexes **26** which were converted into the corresponding rollover complexes **27** by heating (Figure 19) [47]. Whereas the final species can be described as rollover complexes, the reaction is actually a simple metalation, analogous to those displayed by ligands such as 2-phenylpyridine, because it starts forming the monodentate adducts **26**, and represents a case of “pseudo-rollover” cyclometalation. An important difference towards 2-phenylpyridine is the positive charge of the ligand, which becomes neutral after cyclometalation.

At the time, the authors were not able to definitely characterize the cyclometalated complexes **27** as monomeric species excluding dimeric/oligomeric structures; however, in a subsequent paper, the monomeric nature of the complex was defined and a series of derivatives were obtained by means of substitution reactions [48].

The nature of the mesoionic k^2^-N,C- neutral ligand in **27** is interesting and worth to be commented. This ligand belongs to the class of abnormal-remote pyridylene ligand, a well-documented class of *N*-heterocyclic carbenes (NHC) [49,50,51].

**Figure 20:**
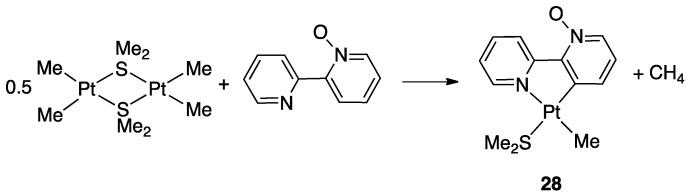
Rollover cyclometalation of 2,2′-bipyridine *N*-oxide. Adapted with permission from *Organometallics* **2014**, *33*, 19, 5402−5413. Copyright (2014) American Chemical Society.

In contrast to the *N*-methyl-2,2′-bipyridylium ion, 2,2′-bipyridine *N*-oxide, **23**, is a neutral ligand with a potential N^O chelating behavior. The Shahsavari group has devoted great attention to the Pt(II) chemistry of this ligand. Cyclometalated rollover complexes of the type [PtMe(k^2^N,C-bipyO-*H*)(L)], **28**, (bipyO-*H*=cyclometalated 2,2′-bipyridine *N*-oxide) were originally obtained by Puddehphatt and coworkers in 2014 [52]. The rollover reaction, in this case, starting from the electron-rich complex [PtMe_2_(μ-SMe_2_)]_2_ is fast even at room temperature, showing that the N-bonded oxygen, at least in the case of Pt(II), strongly favors rollover metalation (Figure 20). As usual, from the starting complex, a family of complexes with different electronic and steric properties were obtained by substitution of the original neutral ligand (SMe_2_). In contrast, the rigid ligand 1,10-phenanthroline *N*-oxide gave the N-O chelated complex.

A few years later, complex **28** was resumed and allowed to obtain a series of complexes of general formula [Pt(N^C)Me(L)], (L = PCy_3_, PPh_2_py, P(OPh)_3_, Ph_2_PCH_2_PPh_2_). The biological activities of these complexes were evaluated against a panel of standard cancer cell lines, and two of them showed a potent cytotoxic activity [53].

In the course of their investigations, Shahsavari and coworkers also studied the oxidative addition reaction of MeI to rollover the Pt(II) N–O bipyridine derived rollover complexes, comparing the rollover complexes with other classical cyclometalated ligands. These results will be reported in Section 4.1.6.

#### 4.1.3. Double Rollover Activation and Dinuclear Complexes (Delocalized Planar Systems)

One peculiarity of rollover cyclometalation derives from the uncoordinated donor atom, which usually is a nitrogen. This nitrogen can coordinate to a second metal center and promote a second rollover metalation, bridging two metals through a highly delocalized, planar connection.

**Figure 21:**
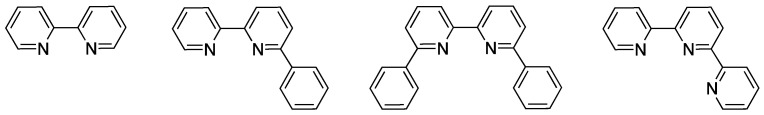
Ligands involved in double rollover cyclometalation.

In addition to 2,2′-bipyridine, other related ligands, such as 6-Ph-2,2′-bipyridine, 6,6′-Ph_2_-bipyridine, and 2,2′:6′,2″-terpyridine (Figure 21) are able to give double rollover metalation, producing planar and highly delocalized complexes. The behavior of these ligands may be complex, due to the presence of several metalation sites.

**Figure 22:**
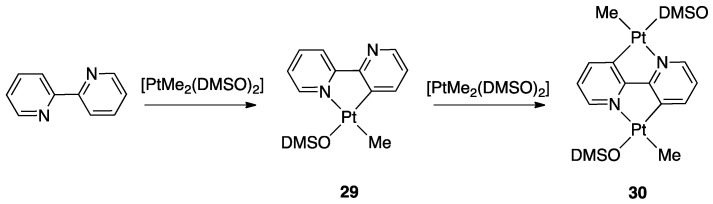
Synthesis of mono- and dinuclear Pt(II) rollover bipyridine complexes.

Reaction of 2,2′-bipyiridine with “PtMe_2_” electron-rich precursors, such as [PtMe_2_(DMSO)_2_], affords the mononuclear complex **29** when the reaction is carried out with 1:1 metal/ligand molar ratio, and dinuclear complex **30** with a 2:1 metal/ligand molar ratio [36]. The mononuclear complex **29** can also be isolated, and then further reacted with [PtMe_2_(DMSO)_2_] (Figure 22). The fact that the reaction can be stopped after the first cyclometalation shows that the second activation step is slower that the first one.

**Figure 23:**
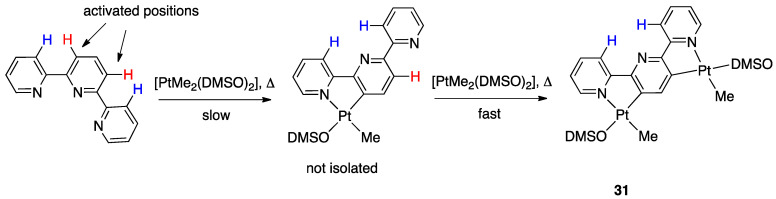
Rollover behavior of 2,2′:6′,2″-terpyridine. In red, activated C–H positions; in blue, not activated C–H positions. Adapted with permission from *Organometallics* **2001**, 20, 6, 1148–1152. Copyright (2001) American Chemical Society.

In contrast, the same reaction with 2,2′:6′,2″-terpyridine (terpy) showed a double C–H rollover activation affording only the binuclear species [(DMSO)MePt(µ-N^C-C^*N*-terpy-2*H*)PtMe(DMSO)], **31** (Figure 23).

Complex **31** is the only reaction product observed both with a Pt/terpy 1:1 and 2:1 molar ratio; when a Pt/terpy 1:1 molar ratio is employed, a mixture of **31** and starting [PtMe_2_(DMSO)_2_] is recovered from the reaction mixture. This means that the second rollover metalation in this case is faster than the first one and, consequently, that the central pyridyl ring after cyclometalation is activated towards rollover metalation, likely due to the higher electron density of the formally anionic pyridyl ligand. In this case, terpyridine, a well-known tridentate ligand, acts as a 2-fold-deprotonated, formally dianionic, highly delocalized planar bridging ligand.

Starting from **31**, substitution of DMSO with neutral ligands afforded a series of derivatives, one of which, [(CO)MePt(μ-N^C-C^*N*-terpy-2*H*)PtMe(CO)] was demonstrated to be an organoplatinum polymer with Pt---Pt interactions, by means of single crystal X-ray structure determination [26,54].

**Figure 24:**
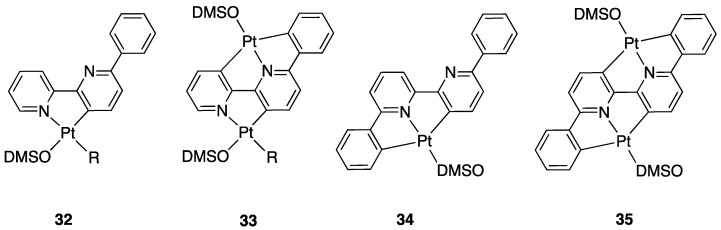
Mono- and dinuclear Pt(II) rollover complexes with phenyl-substituted 2,2′-bipyridines.

At variance, 6-Ph-2,2′-bipyridine showed a similar but more complex behavior [55]. This ligand is well-known for its tendency to give N^N^C classic terdentate Pt(II) cyclometalated complexes by reaction with electron-poor Pt(II) precursors, such as K_2_PtCl_4_. In contrast, by reaction with [PtR_2_(DMSO)_2_] (R = Ph, Me), 6-Ph-2,2′-bipyridine is able to give mono- **(32**) and dinuclear **(33**) complexes (Figure 24). In the latter case, the ligand shows an unprecedented behavior, acting as a threefold-deprotonated ligand. All three Pt–C bonds originate from activation of C(sp^2^)–H bonds.

The dinuclear complex **33** is a unique complexes because (i) the two platinum centers are connected through a ten-electron bridging donor, (ii) three five-membered cyclometalated rings are assembled in the same complex and in same ligand; (iii) the tridentate, formally dianionic, system has a rare C, N, C sequence. From the starting complexes **32** and **33**, a series of novel derivatives were obtained by reaction with neutral ligands or HCl [56].

For comparison, it is worth noting that reaction of 6-phenyl-2,2′-bipyridine with less electron-rich Pt(II) precursors, such as [PtCl_4_]^2−^or *trans*-[PtCl(Me)(SMe)_2_] affords only the adduct [PtCl(Me)(N^N)] or the well-known N^N^C tridentate cyclometalated complex [Pt(N^N^C)Cl].

Extension of the study to 6,6′-Ph_2_-bipyridine gave mono- and dinuclear complexes according to the Pt/ligand molar ratio. With one equivalent of the Pt precursor, the [Pt(L-2*H*)(DMSO)] complex, **34**, is obtained; in this case, the twofold deprotonated ligadopts a C^N^C coordination. The reaction with two equivalents of Pt(II) produces the dinuclear complexes [Pt_2_(L-4*H*)(DMSO)_2_], **35**. In addition, this complex appears unique under several points of view. Its synthesis involves activation of four C–H bonds, with four cyclometalation reactions. In the complex, a fourfold deprotonated 6,6′-Ph_2_-bipyridine (formally tetra anionic) bridges two Pt-DMSO fragments acting as a planar, highly delocalized C^N^C-C^N^C 12-electron donor [57].

**Figure 25:**
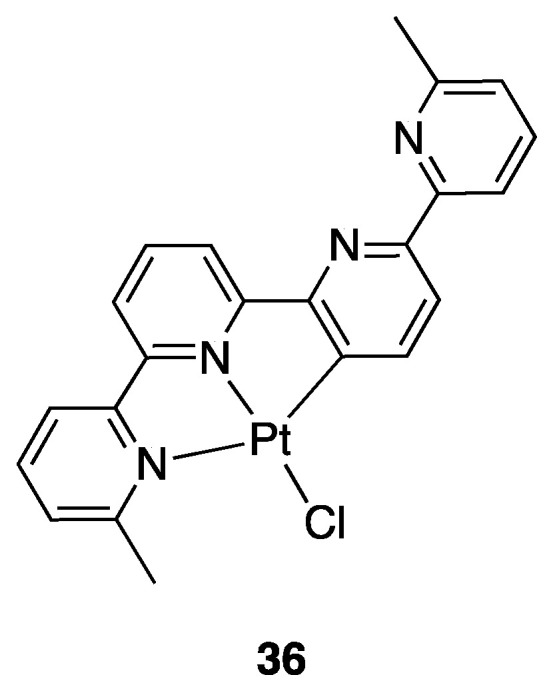
Pt(II) quaterpyridine rollover complex **36**. Reprinted (redrawn) from Polyhedron **2014**, *81*, 188–195. Adamski, A.; Wałȩsa-Chorab, M.; Kubicki, M.; Hnatejko, Z.; Patroniak, V. Absorption spectra, luminescence properties, and electrochemical behavior of Mn(II), Fe(III), and Pt(II) complexes with quaterpyridine ligand. Copyright (2014), with permission from Elsevier.

A fifth analogous ligand, 6,6′′′-Dimethyl-2,2′:6′,2″:6″,2′′′-quaterpyridine, was not studied with “PtR_2_” precursors, but only with PtCl_2_ affording the rollover tridentate complex **36** (Figure 25) [58].

**Figure 26:**
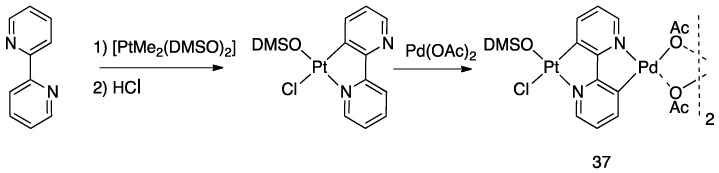
Rollover Pt-Pd heterobimetallic complexes with 2,2′-bipyridine. Adapted with permission from *Organometallics* **2012**, *31*, 8, 2971–2977. Copyright (2012) American Chemical Society.

The double rollover cyclometalation also allowed the build-up of heterobimetallic complexes by means of a two-step reaction sequence e.g., **37**, (Figure 26) [59].

**Figure 27:**
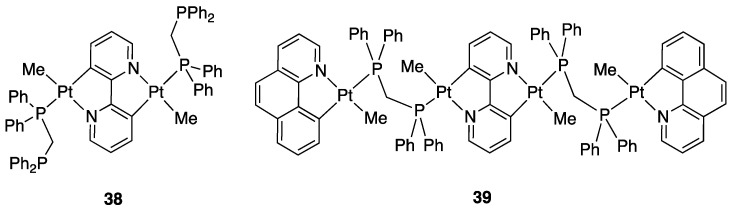
Di- and tetranuclear luminescent rollover complexes **38** and **39**. Reprinted (redrawn) from *J. Organomet. Chem.* **2016**, *819*, 216–227. Aghakhanpour, R.B.; Nabavizadeh, S.M.; Rashidi, M. Newly designed luminescent di- and tetranuclear double rollover cycloplatinated(II) complexes. Copyright (2016), with permission from Elsevier.

In 2016, Aghakhanpour, Nabavizadeh, and Rashidi, following their studies on the chemistry of rollover complexes, synthesized and characterized a series of platinum(II) complexes based on the same “double rollover cycloplatinated core” (Pt(μ-bpy-2*H*)Pt). The authors firstly found that whereas the mononuclear rollover complex [Pt(bpy-*H*)Me(DMSO)], **29**, is completely non emissive in solution and in the solid state, its dinuclear twofold-rollover analogue [Pt_2_(bpy-2*H*)Me_2_(DMSO)_2_] **30** exhibits efficient green emission under the same conditions. The other two complexes, a dinuclear (**38**) and a tetranuclear (**39)** one (Figure 27), also exhibited brightly luminescence in solution and solid state. Complex **30** exhibits a structured emission band, in agreement with the emissive state located in the cyclometalated ligand. Complexes **38** and **39**, however, show unstructured emission bands, indicating a large amount of MLCT in their emissive states [60].

Later, the series of dinuclear complexes was extended, with the synthesis of a series of complexes with the same Pt(μ-bpy-2*H*)Pt core, namely [(L)(L’)Pt(μ-bpy-2*H*)Pt(L)(L)], **40** (L = CF_3_COO, Cl, Br, I; L’ = PR_3_), which showed that the double rollover cycloplatinated core is “a highly versatile and tunable platform for the construction of emissive materials” [61]. The emission behavior of these rollover species was found to be different from that of their correspondent classic cyclometalated complexes, with a high dependence of the emission color on the nature of the ancillary ligands.

#### 4.1.4. Gas Phase Studies

Investigations in the gas phase on rollover reactions, by means of MS spectrometry and DFT computational methods, went ahead in parallel with investigations in solution. It is interesting to observe that the two fields developed almost independently for years. The first studies involving a rollover cyclometalation in the gas-phase were presented by Bursey and coworkers in 1983, which, investigating osmium and ruthenium bipyridine complexes by means of FAB and FD mass spectrometry, reported the formation of [Os(bipy)(bipy-*H*)]^+^ and [Ru(bipy)(bipy-*H*)]^+^ rollover complexes [62,63].

After several years, during which other papers reported results not clearly connected to rollover behaviors, this chemistry was resumed in the period from 2008 to 2012 by H. Schwarz and coworkers, in particular B. Butschke, at the TU Berlin laboratory. Schwarz, Butschke, and coworkers gave a fundamental contribution to the understanding of the rollover reaction, beyond the gas-phase behavior. The Schwarz group extensively studied the rollover chemistry in the gas phase by means of mass spectrometric techniques in conjunction with DFT calculations. Their study also permitted a detailed analysis of the fundamental factors governing the rollover reaction and are well summarized in their 2012 review on the subject [7]. This review provides a quite complete overview of the gas-phase studies, so we will shortly discuss this topic, sending back to this review for an in-depth examination.

Firstly, Schwarz and coworkers investigated the gas-phase behavior of the cationic species [Pt(bipy)((CH_3_)_2_S)]^+^, which loses CH_4_ and (CH_3_)_2_S to give the cyclometalated rollover species [Pt(bipy-*H*)]+ [64].

Labeling experiments, supplemented by DFT computations, shed light into the rollover reaction path in the gas phase: In the course of the reaction, a hydrogen atom from the bipyridine C(3)-position is combined with the coordinated methyl group to form CH_4_. In addition, the thermal reactions of the rollover cation [Pt(bipy-*H*)]^+^ with (CH_3_)_2_S result in an unusual dehydrogenative C-C coupling of the two methyl groups to form C_2_H_4_. The experiments also gave evidence for the reversibility of the rollover process. The conversion of (CH_3_)_2_S to C_2_H_4_ is of interest because it may serve as a model for mechanistic insight in the dehydrosulfurization of sulfur-containing hydrocarbons.

In order to obtain deeper insight into the gas phase behavior, the IMR (ion/molecule reaction) of [Pt(bipy−H)]^+^ with a series of thioethers and thiols was investigated, finding that the rollover-coordinated bipyridine has an active role in hydrogen transfer from the thioether ligand in the course of dehydrosulfurization reaction [65]. The authors conclude that the bipyridine ligand plays an active role, acting as an acceptor in the initial hydrogen transfer from the thioether ligand to the (N^C)Pt^+^ core, a peculiar behavior of rollover cyclometalated complex, not showed by classical analogues.

The study was extended to collision-induced fragmentation in the gas-phase of the cationic complexes [Pt(CH_3_)(L)]^+^ and[Pt(CD_3_)(L)]^+^ (with L = nitrogen-bidentate ligands including 2,2′-bipyridine) and their thermal reactions with a series of deuterium-labeled benzenes. At variance with phenanthroline and bipyrimidine complexes, whose dissociations gave the preferential formation of neutral PtCH_2_ and protonated heterocyclic ligands, the bipyridine complex favors loss of methane through a rollover cyclometalation process ([Pt(bipy)CH_3_]^+^ ➝ [Pt(bipy-*H*)]^+^ + CH_4_) [66].

The gas-phase studies continued with the investigation on the ion–molecule reactions of rollover-cyclometalated [Pt(bipy-*H*)]^+^ with alkyl ethers in the gas-phase, in comparison with corresponding reactions with sulfur analogues [67,68]. Furthermore, the gas-phase ion/molecule reactions of the cationic cyclometalated species [Pt(bipy–H)]^+^ with chloromethanes CH_4-n_Cl_n_ (n = 1–4), lead to a platinum-mediated C–C bond formation [69].

Investigations on thermal ion/molecule reactions of a series of cyclometalated Pt(II) complexes [Pt(L–H)]^+^ (L = 2,2′-bipyridine, 2-phenylpyridine (phpy), 7,8-benzoquinoline (bq)) with branched and linear alkanes showed that only the rollover complex undergo hydrogen-atom transfer from the alkane to the coordinated carbon of the cyclometalated pyridine, followed by ring rotation. DFT calculations were used to elucidate reaction pathways and effects of different ligands on the course of the reactions [70]. The main processes correspond to elimination of H_2_ and alkenes. For all three cyclometalated complexes, loss of C_2_H_4_ from C_2_H_6_ dominates over H_2_ elimination; however, the mechanisms of the rollover complex significantly differ for the reactions of classical cyclometalated complexes. In the case of the rollover complex, a double hydrogen atom transfer from C_2_H_6_ to the cationic [Pt(bipy-*H*)]^+^ is followed by internal ring rotation in a retro-rollover process, affording [Pt(H)(bipy)]^+^, for the phpy and bq complexes, the cyclometalated pattern is preserved to give [Pt(H_2_)(L–*H*) species. This observation indicates that different reaction mechanisms are operating in the dehydrogenation of C_2_H_6_: only the (bipy-*H*) rollover ligand can return to a bidentate N, N′-coordination mode after the hydrogen transfer, following a retro-rollover pathway.

In a systematic investigation, a comparison of the gas-phase fragmentation of nickel, palladium, and platinum [M(bipy)X]^+^ cationic complexes (X = CH_3_, F, Cl, Br, I, OAc) showed that upon collision-induced dissociation (CID), only the platinum species [Pt(bipy)CH_3_]^+^ and [Pt(bipy)Cl]^+^ gave rollover cyclometalation, whereas [Pd(bipy)CH_3_]^+^ and [Ni(bipy)CH_3_]^+^ gave homolytic cleavage of metal-CH_3_ bond. On the whole, the observations suggest that platinum is superior to palladium and nickel for rollover metalation. Mechanistic considerations, based on experimental and DFT data, were reported in Section 3 [21].

Gas-phase studies were also performed with the intention to shed light into the rollover catalytic activity of an (h^6^-arene)RuCl(N^N) complex (N^N = 2-(pyrimidin-4-yl)pyridine-type ligands) [71,72]. The investigation combined gas phase, solution, and in-silico studies will be thoroughly discussed in Section 5 (see Section 5.1).

**Figure 28:**
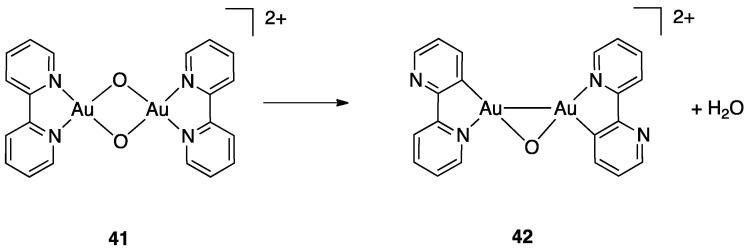
Rollover Au(III) cyclometalation in the gas-phase with loss of water. Adapted with permission from *J. Am. Chem. Soc.* **2009**, *131*, 36, 13009–13019. Copyright (2009) American Chemical Society.

A further example of rollover behavior in the gas-phase involved the fragmentation of the dinuclear gold m-oxo complex **41**, which liberates H_2_O by means of a twofold rollover cyclometalation affording the cationic complex **42**. (Figure 28) [73].

#### 4.1.5. Protonation and Retro-Rollover

**Figure 29:**
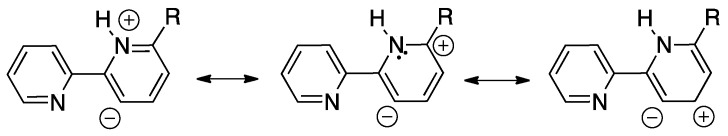
Resonance structures of the mesoionic (or abnormal-remote pyridylene) ligand bipy*.

**Figure 30:**
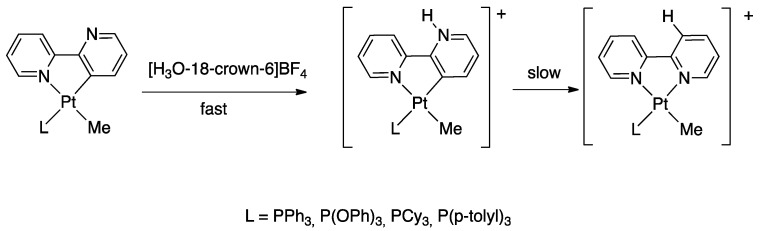
The retro-rollover process. Adapted with permission from *Organometallics* **2013**, *32*, 2, 438–448. Copyright (2013) American Chemical Society.

As commented before, the presence of an uncoordinated nitrogen atom in 2,2′-bipyridine rollover complexes deeply influences the reactivity of these complexes. In particular, the nitrogen atom can be protonated affording new cationic complexes where the mesoionic neutral ligand bipy* (Figure 29 and Figure 30) is an isomer of 2,2′-bipyridine and can also be described as abnormal-remote pyridylene [49,50,51].

Protonation of [Pt(bipy-*H*)Me(L)] complexes (L = DMSO, PPh_3_, PCy_3_, etc.) with [18-crown-6-H_3_O][BF_4_] produced a series of stable pyridylenes [Pt(bipy*)(Me)(L)]+. These cationic species in solution follow a retro-rollover reaction, affording the corresponding N^N chelated complexes [Pt(bipy)(Me)(L)]^+^ (30). The retro-rollover process corresponds to an isomerization process; the reaction proceeds at different rates depending on the nature of the phosphane ligand, with the most basic PCy_3_ providing the fastest reaction. The mesoionic species [Pt(bipy*)(Me)(L)]^+^ contain two Pt−C bonds: the balance between the Pt−C(sp^2^) and Pt−C(sp^3^) bond rupture is subtle, and competition is observed in the presence of strong external donors [74].

##### Ligands with Multiple Personalities

**Figure 31:**
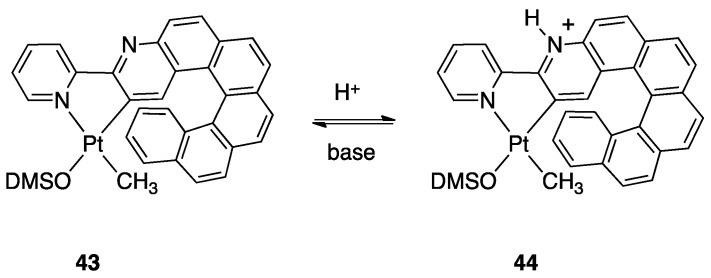
Rollover organometallic helicenes. Adapted (redrawn) with permission from *Chem. A Eur. J.* **2015**, *21*, 1673–1681. Copyright (2015) John Wiley & Sons, Inc.

Protonation and retro-rollover processes of Pt(II) rollover complexes were also achieved with other related ligands, such as 2-(2′-pyridyl)quinoline, 6-methoxy)-2,2′-bipyridine, and the helicene-2,2′-bipyridine proligand 3-(2-pyridyl)-4-aza[6]helicene (Figure 31). The rollover complex **43**, obtained by reaction with [PtMe_2_(DMSO)_2_], reacts with HBF_4_ affording the corresponding protonated species **44** [75]. The protonation reaction is reversible due to acid/base switching and both the complexes, described as organometallic helicenes, that act as “multifunctional switchable systems” due to the reversible tuning of optical and chiroptical properties: optical rotation, electronic circular dichroism, nonpolarized luminescence, and circularly polarized luminescence.

#### 4.1.6. Reactivity of Rollover Complexes

Although beyond the scope of this Review, we will briefly comment some aspects of the reactivity of bipyridine Pt(II) rollover complexes. One important aspect of the reactivity of [Pt(bipy-*H*)Me(L)] rollover species (L = neutral ligand) is their manifold reactivity with electrophiles, due to the presence of several nucleophilic centers or bonds: the uncoordinated nitrogen, the electron-rich platinum center, and the two Pt-C bonds. The reactivity with acids has been described in Section 4.1.5, being connected with the retro-rollover process. The reaction with alkyl halides usually leads to oxidative additions to afford Pt(IV) complexes. This reactivity is also related to functionalization of the bipyridine ligands and will be treated in Section 5.2.

Starting from the parent complex [Pt(bipy-*H*)Me(DMSO)], a series of rollover complexes with different electronic and steric properties have been obtained by substitution of the neutral and anionic ancillary ligands. The structural modifications allow the obtainment of species with an ample variety of properties and potential applications, from emissive materials to antitumor drugs.

**Figure 32:**
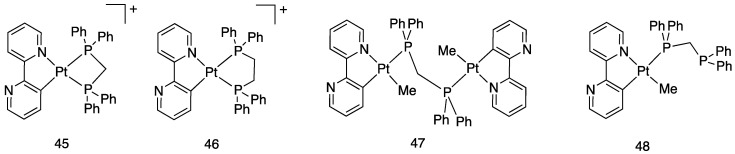
Examples of mono- and dinuclear Pt(II) rollover complexes complexes of 2,2′-bipyridine and 2,2′-bipyridine *N*-oxide with diphosphines compounds **45**–**48**. Ref. [76]—Reproduced (redrawn) by permission of The Royal Society of Chemistry.

Some mono- and dinuclear complexes with diphosphines have been reported earlier in this review. Other series of mono, and dinuclear complexes with diphosphines, **45**–**48** (see Figure 32), have been reported by us [76] and other researchers [77,78], comprising the bipyridine *N*-Oxide complex [Pt(N^C)(dppm)(p-tolyl)], **49**, analogous to **48**, with p-tolyl in place on methyl.

Several of these complexes may be obtained from the parent rollover compounds [Pt(bipy-*H*)Me(DMSO)] and [Pt_2_(μ-bipy-2*H*)Me(DMSO)] by substitution reaction of the neutral ligand or by reactivity of the coordinated methyl (e.g., by reaction with HCl). Among the ample family of the described complexes, some have interesting properties, such as the dinuclear double-rollover complexes **40**, [Pt_2_(μ-bpy-2*H*)(X)_2_(PPh_3_)_2_], whose emission properties dependson the nature of the anionic ligand X (e.g., halide or trifluoroacetate) [79]. The authors proved that the halide ligands tune the brightness of these compounds as “organic light emitting diodes (OLEDs) emitters”.

Finally, we may also mention auto-assembly of an unusual polynuclear rollover hydride [Pt(N^C)(μ-H)]_4_, obtained by reaction of [Pt(bipy-*H*)Me(DMSO)] with NaBH_4_ [80].

#### 4.1.7. Other Metals

The iridium rollover chemistry is rather rich; as previously shown, this metal has been one of the first to display rollover metalation, and the first one with 2,2′-bipyridine. After the final characterization of [Ir(bipy)_2_(bipy*)]^3+^, it took five years until a second iridium rollover complex, [Ir(bipy)(bipy-*H*)Cl]_2_^2+^, was obtained by reaction of Ir(IV) and Ir(III) chlorides with 2,2′-bipyridine under controlled conditions [81].

**Figure 33:**
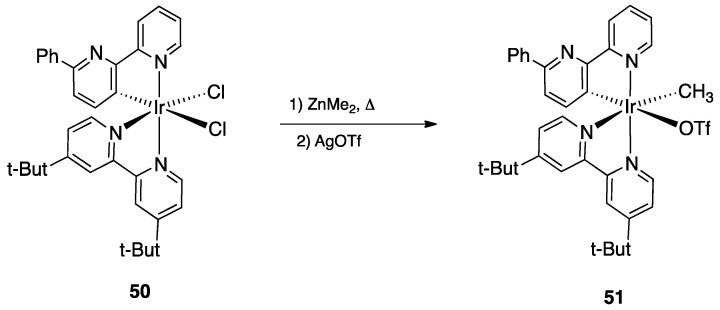
Synthesis of complex **51**. Adapted with permission from *Organometallics* **2007**, *26*, 9, 2137–2140. Copyright (2007) American Chemical Society.

An interesting case of Ir(III) rollover complex, whose reactivity have implications in organic synthesis and catalysis, was reported by Periana and coworkers in 2007. Reaction of 6-phenyl-2,2′-bipyridine with IrCl_3_ and, subsequently, with 4,4′-di-*tert*-butyl-2,2′-bipyridine, afforded the rollover complex **50**, from which successive reactions with ZnMe_2_ and AgOTf allowed isolation of the rollover complex **51** (Figure 33) [82].

Complex **51** showed interesting reactivity which comprehends both stoichiometric and catalytic processes. As for stochiometric reactivity, the Ir(III) complex promotes C–H bond activation in benzene, generating the corresponding Ir-phenyl complex. In addition, reaction with oxidants such as bis(trifluoroacetate)iodobenzene resulted in oxy functionalization of the coordinated alkyl group at room temperature. According to the authors, this was the first example of “relatively efficient Ir(III)-alkyl functionalization to generate oxy functionalized products”. Furthermore, this rollover Ir(III) complex also showed to be active in a catalytic process promoting C–H activation in benzene in the presence of acids.

**Figure 34:**
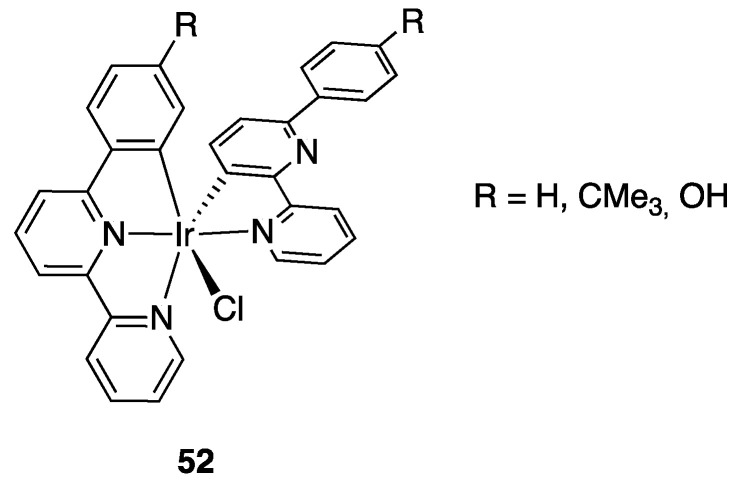
Ir(III) rollover complex **51**. Adapted with permission from *Organometallics* **2009**, *28*, 12, 3395–3406. Copyright (2009) American Chemical Society.

Two years later, the study was extended to three related tridentate ligands 6-(4-R-phenyl)-2,2′-bipyridine, where (R = H, CMe_3_, OH). The ligands gave easily rollover cyclometalation by reaction with IrCl_3_, affording the dinuclear complexes [Ir(N^C)Cl_2_(C_5_H_5_N)]_2_ or, with a different metal to ligand molar ratio, the bis-cyclometalated complexes **52**, [Ir(N^N^C)(N^C)Cl] where the 6-Ph-bipyridine ligands act both as classical tridentate and rollover bidentate cyclometalating ligand (Figure 34). From the dinuclear complex with 6-Ph-bipyridine the corresponding mononuclear complex Ir(N^C)(N^N)Cl_2_ was obtained by reaction 4,4′-di-*tert*-butylbipyridine [83].

**Figure 35:**
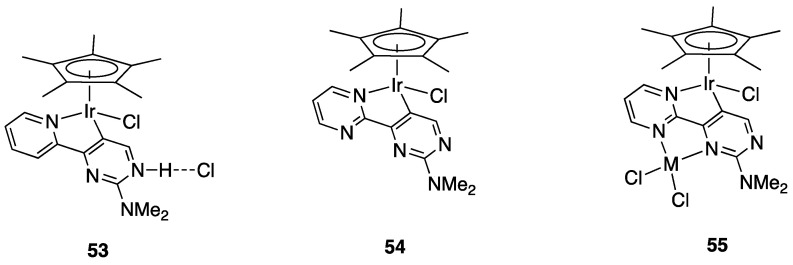
Ir(III) rollover complexes **53** (Reproduced (redrawn) from Ref. [27] with permission from the Centre National de la Recherche Scientifique (CNRS) and The Royal Society of Chemistry), **54** and **55** (both reproduced (redrawn) from Ref. [84] with permission from The Royal Society of Chemistry).

Years later, Niedner-Schatteburg, Thiel, and coworkers reported the rollover behavior of the iridium(III) dimer [(η^5^-Cp*)IrCl(μ-Cl)]_2_ with 2-(2-dialkylaminopyrimidin-4-yl)pyridine whose rollover cyclometallation proceeds even at room temperature affording complex **53** (Figure 35).

In order to get mechanistic insights, the reaction of Co, Rh, and Ir precursors [(η^5^-Cp*)MCl(μ-Cl)]_2_, with five different 2-heteroarylpyridines was studied in the gas-phase. As reported in Section 6, the “RhCp*” complex has been widely used as catalysts for numerous rollover C–H bond functionalization of aromatic and heteroaromatic compounds.

In the course of the study, cationic adducts [(h^5^-Cp*)M(Cl)(N,N′)]^+^ with 2-(2-dialkylaminopyrimidin-4-yl)pyrimidine (M = Co, Rh, Ir) were examined in the gas phase by DFT calculations and CID ESI-MS spectrometry, displaying a rollover cyclometallation at one of the aromatic rings to give complexes analogous to **53**. As for the metal influence, rollover cyclometallation proceeds easier from cobalt to rhodium to iridium: the trend follows the stabilities of the metal–carbon bonds formed [27].

The Ir(III) rollover complex **53** has, as usual, an *ortho* nitrogen available for coordination. This presence gave the idea to extend the study to the analogous pyrimidine ligand, 2-(2-dialkylaminopyrimidin-4-yl)pyrimidine. The resulting rollover complex **54** was used to obtain the heterobimetallic Ir-Pt and Ir-Pd complexes **55** by reaction with [(PhCN)_2_MCl_2_] or K[Pt(C_2_H_4_)Cl_3_] [84].

**Figure 36:**
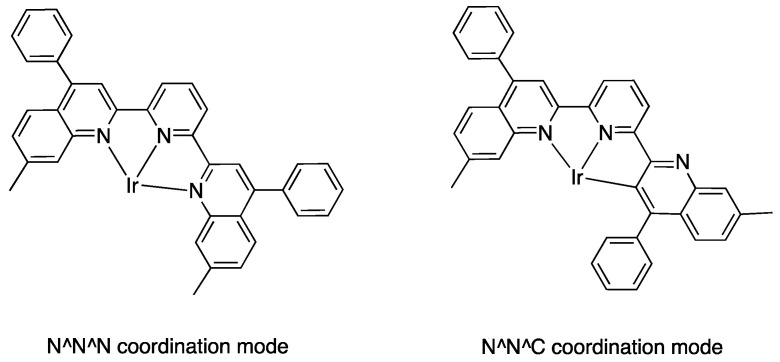
N^N^N and N^N^C coordination modes of 2,6-bis(7′-methyl-4′-phenyl-2′-quinolyl)pyridine with Ir(III).

Ir(III) derivatives where also used to obtain other bipyridine- or terpyridine-based rollover complexes. The potentially N^N^N terdentate ligand 2,6-bis(7′-methyl-4′-phenyl-2′-quinolyl)pyridine reacts with IrCl_3_ in glycerol at reflux, coordinating either in the classical N^N^N mode or as an N^N^C ligand (Figure 36) as a consequence of a rollover cyclometallation of one lateral pyridine ring. In particular, the author obtained the bis-tridentate complexes, [Ir(N^N^N)(N^N^C)]^2+^ and [Ir(N^N^C)_2_]^+^. Inter alia, the new complexes showed to be the first luminescent and redox-active Ir(III)-cyclometalated bis-tridentate complexes [85].

**Figure 37:**
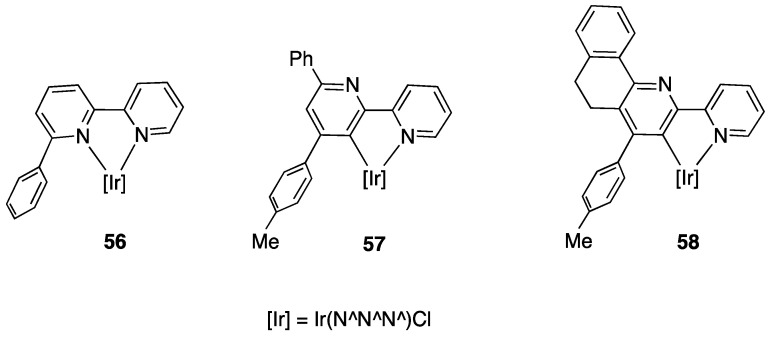
Ir(III) complexes with 6′-aryl-2,2′-bipyridines.

Years later, Bexon and Williams studied the reaction of iridium terpyridyl trichloride, [Ir(N^N^N)Cl_3_], (N^N^N = terpyridine) with three potentially terdentate N^N^C ligands with the 6′-phenyl-2,2′-bipyridine scaffold. Under the reaction conditions used, none of the three ligands showed tendency to act in the classic cyclometalated N^N^C mode (Figure 37). Likely due to the steric hindrance of the terpyridine ligand, 6′-phenyl-2,2′-bipyridine binds to the metal in a simple N^N coordination mode (complex **56**), whereas the 4′-tolyl groups in the other two ligands promotes a rollover C–H bond activation, affording complexes **57** and **58** (Figure 37) [86]. Interestingly, the two new rollover complexes exhibit intense luminescence in the solution at room temperature.

Rollover cyclometalation of 4,4′-Me_2_-2,2′-bipyridine has been reported by means of a one-pot reaction with IrCl_3_ and a bis-trifluoromethyl-phenylpyridine (2-(3,5-bis(trifluoromethyl)phenyl)-4-methylpyridine), followed by treatment with lithium 2,4-pentanedionate. The reaction afforded three products, separated by column chromatography. The rollover complex [Ir(N^C)(N^′C′)(acac)], **59**, (N^C = cyclometalated 2-(3,5-bis(trifluoromethyl)phenyl)-4-methylpyridine, N′^C′ = rollover cyclometalated 4,4′-Me_2_-2,2′-bipyridine) was isolated albeit with a low yield, and characterized by means of X-ray crystallography [87].

**Figure 38:**
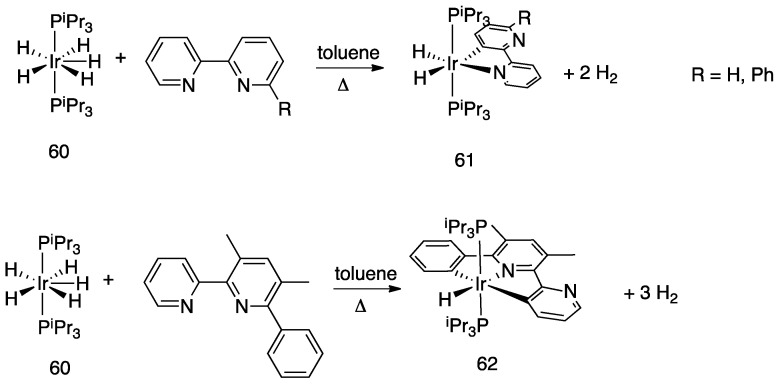
Synthesis of rollover complexes **61** and **62**. Adapted with permission from *Organometallics* **2020**, *39*, 11, 2102–2115. Copyright (2020) American Chemical Society.

Iridium and osmium polyhydrides were also found to be able to promote rollover C–H bond activation in 2,2′-bipyridines and related heterocycles [28]. Reaction of the iridium(V) hydrido complex **60** with 2,2′-bipyridine or 6-Ph-2,2′bipyridine in refluxing toluene leads to rollover C–H bond activation, allowing isolation of complexes **61** (Figure 38).

Reaction of 6-phenyl-2,2′-bipyridine with the pentadeuteride complex [IrD_5_(PR_3_)_2_] reveals that the generation of the rollover complex can be rationalized as a pyridyl-assisted process, as the corresponding osmium complexes that will be described in Section 4.1.7.

In contrast, when the internal pyridine ring is protected, as in 3,5-dimethyl-6-phenyl-2,2′-bipyridine, the reaction resulted in rotation of the external pyridine ring, with a two-fold metalation, affording the C, N, C- rollover-pincer complex **62** (Figure 38).

##### Osmium

**Figure 39:**
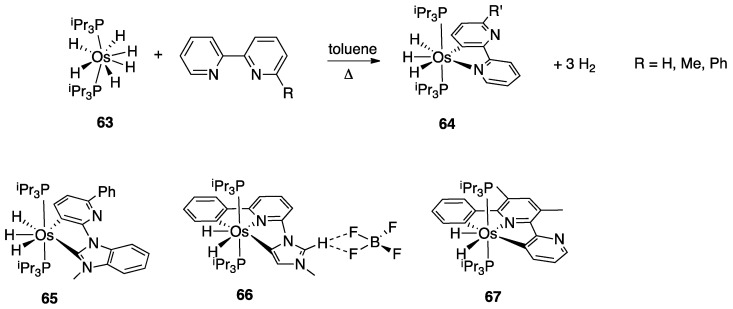
Os(IV) rollover complexes. Adapted with permission from *Organometallics* **2020**, *39*, 11, 2102–2115. Copyright (2020) American Chemical Society.

In the same paper, reaction of the hexahydride osmium complex **63** with 2,2′-bipyridine or 6-substituted 2,2′-bipyridines (Ph and Me substituents) under reflux results, as the analogous Ir(V) complexes, in rollover C(3)–H bond activation and formation with high yields of complexes **64** (Figure 39). Mechanistic studies, carried out with the corresponding polydeuteride Os(VI) complex, indicates that the C–H bond activation is not a N-directed process and precedes nitrogen coordination. The coordinatively saturated complex **63** needs to release a hydrogen molecule, through a reductive elimination process, before reacting with the C–H bond. The resulting electrophilic Os(IV) center promote the hydride-mediated heterolytic cleavage of the C–H bond. The C–H bond selectivity is considered the result of nitrogen trapping of the intermediate formed by C–H activation on the other ring [29].

Other related heterocycles were investigated with interesting results. Reaction with 3-methyl-1-(6-phenylpyridin-2-yl)-1*H*-benzimidazolium tetrafluoroborate proceeded in a similar way affording complex **65**. In contrast, modification in the ligands scaffolds gives rise to a drastic change in the behavior. When the benzimidazolium group is replaced by imidazolium, the absence of protection in the five-membered ring allows its metalation in an abnormal position, producing the C^N^C complex **66**. Analogously, protection of the position 3 of 6-phenyl-2,2′-bipyridine with a methyl group drives rollover C–H activation to the external pyridine ring producing the two-fold deprotonated C^N^C complex **67**.

A few other cases have been reported for rhodium, ruthenium, copper, gold, and rhenium. In 2006, Zuber and Pruchnik showed that also rhodium might be involved in a rollover C–H activation process, finding that a solution of Rh^I^(bipy) complexes showed H/D exchange at the C(3) and C(3′) positions. The observation of hydride signals in the ^1^H-NMR spectrum strongly suggests oxidative addition of bipyridine C(3)-H to rhodium through a rollover pathway, to form the Rh^III^ hydrido complex [Rh(N^N)(N^C)(H)(CD_3_O)], **68**, having one chelated N^N and one rollover N^C bipyridine ligand [88].

Rh(III) is also able to promote double rollover activation on both the pyridine rings of 2,2′-bipyridine, as shown by heating the dicationic rhodium(III) complex [Rh(bipy)(CH_3_)(H_2_O)_3_][BF_4_]_2_ to give the solvato species [Rh(C^C)(CD_3_)(H_2_O)_3_], with a double-rollover C(3)^C(3′) twofold deprotonated bipyridine, **69** [89]. The same complex, at room temperature, promoted incorporation of deuterium in the coordinated methyl group.

**Figure 40:**
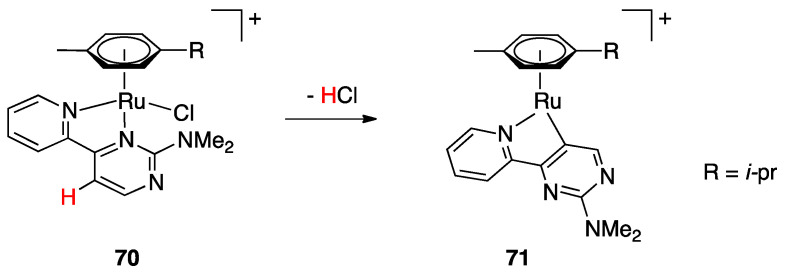
Switch from N^N to N^C coordination mode promoted by a Ru(II) complex. Adapted from *Eur. J. Inorg. Chem*. **2013**, 4305–4317.

An interesting case appeared in 2013, when Niedner-Schatteburg, Thiel, and coworkers reported the synthesis of the air stable Ru(II) complex **70**, which, in the course of catalytic transfer hydrogenation of ketones showed to be able to switch the bidentate ligand from N^N (neutral) to rollover N^C (anionic, deprotonated) coordination mode, activating a C–H bond in the pyrimidine ring (Figure 40). The self-rollover activation of complex **70** to give **71** placed the basis for a hydrogen transfer process, which was investigated by the authors and will be discussed in Section 5 [90].

Copper entered in the rollover-family in 2008 when Yang and coworkers reported the double rollover metalation of 2,2′-bipyridine in a dodeca-copper cluster. In the cluster, the bipy-2*H* group acts as a twofold-deprotonated anionic N^C-C^N ligand, bonding two Cu^II^ atoms [91].

**Figure 41:**
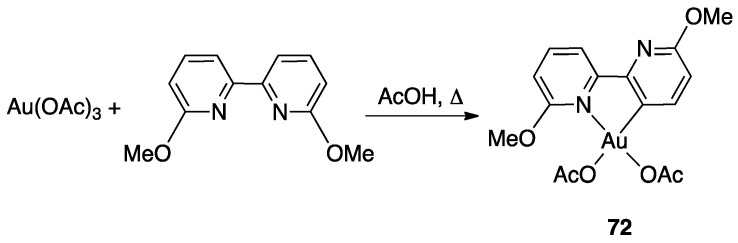
The first (and only) gold rollover complex. Adapted with permission from *Organometallics* **2010**, *29*, 5, 1064–1066. Copyright (2010) American Chemical Society.

Although several bipyridine ligands were tested with gold(III) derivatives, only 6,6′-dimethoxy-2,2′-bipyridine was activated by means of a rollover pathway, affording, by reaction with gold(III) acetate, the rollover complex **72** by direct C–H bond activation in acetic acid (Figure 41) [92].

**Figure 42:**
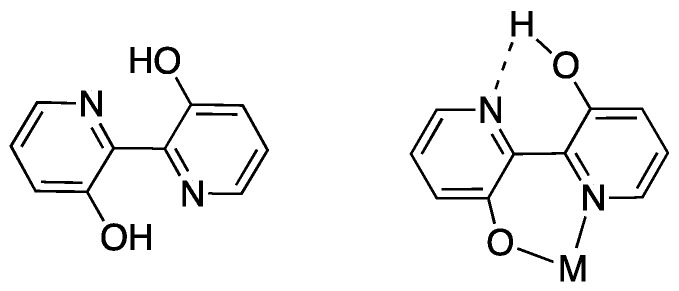
3,3′-dihydroxo-2,2′-bipyridine (left) and N^O coordination mode in complex **73** (right).

The last example in this series of complexes regards rhenium and is a case of “pseudo” rollover pathways, not including C–H bond activation. Deprotonation of the rhenium complex [ReCl(N^N)(CO)_3_] (N^N = 3,3′-dihydroxo-2,2′-bipyridine), followed by exposure to light, affords the anionic complex [Re(N^O)(CO)_3_Cl] **73** (N^O = deprotonated 3-hydroxo,3′-oxo-2,2′-bipyridine, see Figure 42) [93]. The system was recently used in the catalytic reduction of carbon dioxide [94].

### 4.2. Ligands Other than Bipyridine

2,2′-bipyidines can be considered as the prototypical rollover ligands; however, a series of other ligands, mostly hererocyclic bidentate donors, have been reported as well.

#### 4.2.1. Platinum and Palladium Complexes

**Figure 43:**
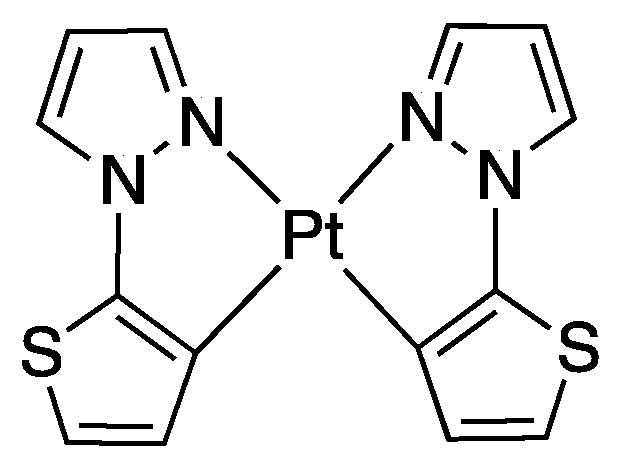
Complex **74**.

As described in Section 2, the first rollover complexes regarded a non-bipyridine ligand, involving 2-(2′-thienyl)pyridine Pt(II) and Pd(II) complexes [9]. Years later, in 1987, Chassot and von Zelewsky reported the synthesis of a series of homoleptic Pt(II) cyclometalated complexes with aromatic ligands, one of which, **74**, derived from 1-(2-thienyl)pyrazole (Figure 43). The reaction is actually a pseudo-rollover process, since it does not involve a C–H bond activation, but requires reaction of *trans*-PtCl_2_(SEt_2_)_2_ with the organolithium derivative 5-(1-2-thienyl)pyrazolyl))lithium [95].

**Figure 44:**
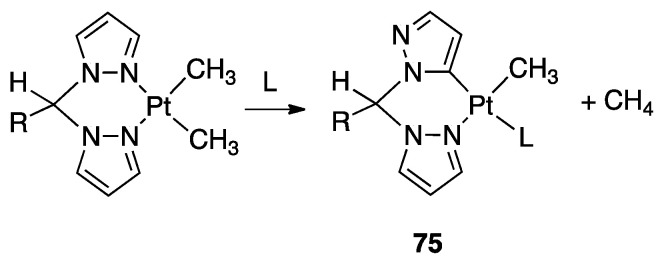
Rare Pt(II) 6-membered rollover cyclometalated complexes.

An interesting rollover cyclometalation involving pyrazole rings was reported a few years later. A series of dimethyl platinum(II) complexes [PtMe_2_(N^N)] with several polydentate nitrogen ligands containing pyrazoles underwent rollover cyclometalation when dissolved in pyridine at room temperature (R = H, Ph, pyrazole, or *N*-methylimidazol-2-yl, Figure 44). The resulting complexes **75** (L = py, PPh_3_, CO, etc.) represent rare cases of six-membered cyclometalated rollover rings. From the starting Pt(II) derivatives, oxidative addition reactions with organohalides afforded the corresponding Pt(IV) rollover complexes [96].

**Figure 45:**
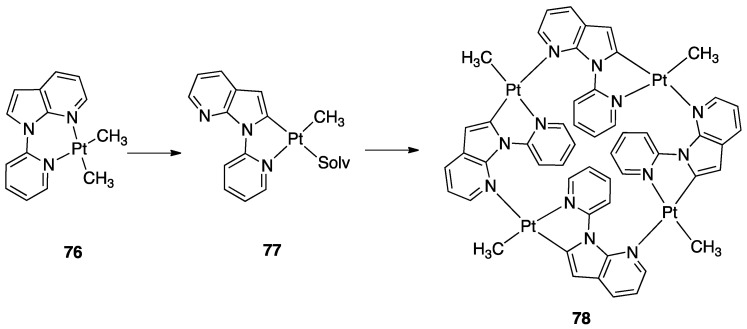
Self-assembly room-temperature formation of tetramer complex **78.** Adapted with permission from *J. Am. Chem. Soc*. **2007**, *129*, 11, 3092–3093. Copyright (2007) American Chemical Society.

Non-bipyridine rollover complexes are rare. It took almost twenty years until a new Pt(II) case was reported in the literature. In an elegant work, Wang and coworkers described an example of room temperature rollover C–H bond activation promoted by Pt(II) (Figure 45). The adduct **76** is stable in the solid state at ambient temperature, but only under 5 °C in solution. For this reason, from the six-membered N^N chelated complex, a five-membered rollover cyclometalated N^C complex was easily formed. Likely due to the not exceptional stability of **76**, the Pt–N bond rupture is fast and reversible. In the presence of a good coordinating solvent such as CH_3_CN, the mononuclear rollover complex **77** (S = CH_3_CN) was isolated and characterized by X-ray diffraction.

The authors proposed an oxidative-addition reductive-elimination pathway for the process, based on the electron-richness of the Pt center in the three-coordinate intermediate. In THF, CH_2_Cl_2_, or benzene solution, a “roll-over cyclometalation driven self-assembly process at ambient temperature” afforded the tetramer complex **76** (Figure 45). The tetramer assembly was promoted by coordination of the free nitrogen atom in the rollover mononuclear complex, a typical feature of rollover complexes, which easily leads to dinuclear species. The rollover complex **78** was fully characterized by means of NMR, elemental analysis, and X-ray diffraction [24].

On the basis of common mechanisms of cyclometalation reactions, the authors proposed an initial Pt–N bond breaking step which generates a three-coordinate rollover complex. The electron-rich Pt(II) center promotes the oxidative addition of the *ortho*-C–H bond on the azaindole to give a rollover Pt(IV) hydride intermediate, from which reductive elimination of methane gave the solvato species **75** that spontaneously self-assembles to the cyclic Pt_4_ compound **78**.

Kinetic NMR analyses established a first-order decay of the adduct [Pt(N^N)Me_2_] with time in good agreement with an intramolecular cyclometalation process; the C–H rollover cleavage was found to be the rate determining step of the overall process.

**Figure 46:**
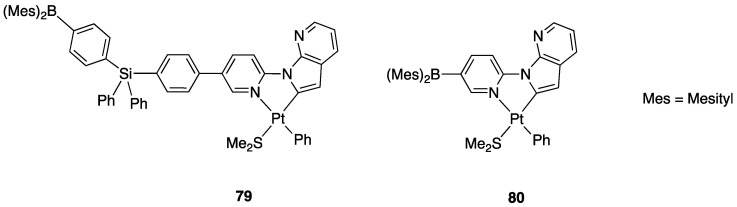
Rollover complexes **79** and **80**. Adapted from *Chem. A Eur. J.* **2009**, *15*, 6131–6137.

Successively, the same group reported the rollover metalation of related triarylboron ligands. Additionally, in this case, rollover cyclometalation occurs very easily by reaction with Pt(II) electron rich precursors, affording the corresponding cyclometalated complex **79** and **80** (Figure 46). The complexes exhibit interesting phosphorescent properties, with unusually long phosphorescent decay time. Addition of fluoride was found to result in a large enhancement of the phosphorescent emission intensity of the complexes. However, complex **80** is not dual emissive as **79** and shows only metal-to-ligand change-transfer-based phosphorescence [97].

**Figure 47:**
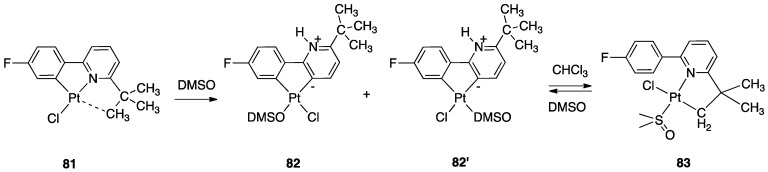
Switchable rollover-classical cyclometalation reaction driven by the solvent. Adapted with permission from *Organometallics* **2011**, *30*, 13, 3603–3609. Copyright (2011) American Chemical Society.

An uncommon case of rollover cyclometalation, resulting in a C^C metallacycle, was reported by Rourke and coworkers. Starting from the 14-electron tricoordinated Pt(II) complex **81**, stabilized by an agostic Pt---_3_HC interaction, the unusual isomeric rollover complexes **82** and **82′** were slowly formed at ambient temperature in DMSO. Starting from **82** and **82′**, a switchable reaction, driven by the solvent, was observed: in CHCl_3_, the rollover complex was transformed into the classical cyclometalated complex **83**, whereas in the more polar solvent DMSO, the reverse reaction occurs regenerating the rollover complex (Figure 47). These data have been explained in terms of a delicate balance (induced by the bulkiness of the *tert*-butyl group), controlled by solvent polarity [98].

**Figure 48:**
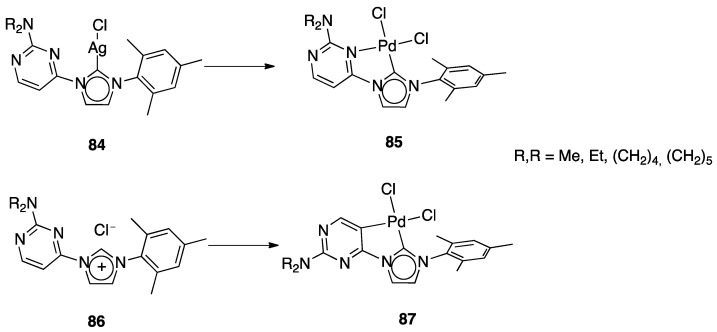
NHC-rollover palladium complexes. Adapted from *Chem. Eur. J.* **2017**, *23*, 14563–14575.

Another unusual and very interesting case, regarded an NHC-rollover, C^C chelated, Pd(II) complex, reported by Thiel and coworkers starting from the imidazolium salts **86** (Figure 48).

Depending on the reaction conditions, both C^N and C^C complexes were obtained (complexes **85** and **87**). Two routes were used for the two classes of complexes: transmetalation with the corresponding Ag-NHC complexes **84** gave the C^N cyclopalladated complexes **85**, whereas reaction of PdCl_2_ with the imidazolium salts produced the C^C rollover complexes **87** [99].

**Figure 49:**
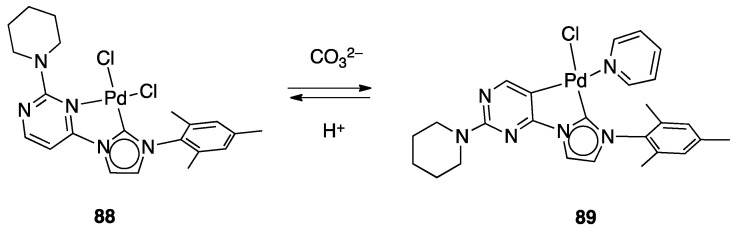
Reversible rollover reaction of the NHC-rollover palladium complex **89**. Adapted from *Chem. Eur. J.* **2017**, *23*, 14563–14575.

The rollover cyclometalation reaction is partially reversible. Treatment of the N^C complex **88** with potassium carbonate in deuterated pyridine at 80 °C gave complex **89** with a 95% conversion (NMR criterion), whereas treatment of **89** with HCl gave evidence of the presence of complex **88** (along with an intermediate specie), eventually resulting in decomposition (Figure 49).

The new rollover compounds showed high catalytic activities in Suzuki–Miyaura cross-coupling reactions. Catalytic processes involving rollover complexes will be treated in Section 5 and Section 6.

As we are seeing, rollover cyclometalated complexes may appear in different subclasses. In addition to bidentate N^C and C^C derivatives, another interesting subclass is that of pincer-rollover complexes. This behavior originates from the fact that some polydentate ligands are able to switch between multiple coordination modes. A first example of pincer-rollover coordination involved the potentially tridentate ligands **90**–**92**, defined “pincer click ligands” (PCL) due to their coordinative versatility.

**Figure 50:**
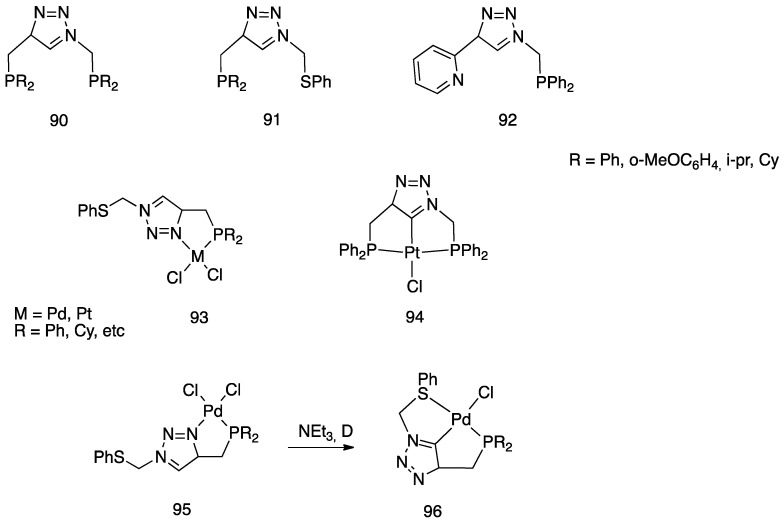
Pincer ligands **90**–**92** and examples of bidentate (**93**), pincer tridentate (**94**), and rollover tridentate (**96**) complexes. Adapted with permission from *Organometallics* **2009**, *28*, 24, 7001–7005. Copyright (2009) American Chemical Society.

Under a choice of reaction conditions, these ligands can selectively behave as pincer tridentate or rollover bidentate ligands both with Pd(II) and Pt(II) metal precursors (Figure 50). The authors were able to demonstrate a “rollover switch” of kinetically formed products (i.e., rollover bidentate complexes) to the thermodynamically favored products (i.e., tridentate pincers) [100]. As an example, the authors were able to “rollover switch” the P^N bidentate complexes **95**, kinetically controlled, to the tridentate complex **96**, which is the thermodynamically favored species (Figure 50).

A second case of pincer-rollover switch regarded the behavior of two unsymmetrical phosphine-pyrazole P^C^N pincer ligands (P^C^N = 1-[3-[(di-*tert*-butylphosphino)methyl]phenyl]-1*H*-pyrazole, and its methylated analogue, P^C^N-Me) **97** and **98**).

**Figure 51:**
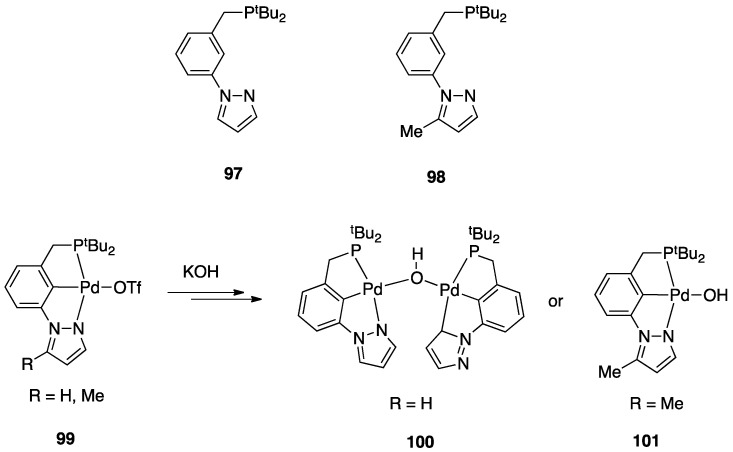
Pincer ligands **97** and **98** and rollover P^C^C tridentate Pd(II) complex **100**. Adapted with permission from Organometallics **2015**, *34*, 16, 3998–4010. Copyright (2015) American Chemical Society.

In the course of the synthesis of palladium(II) complexes, starting from complex **99**, the monomeric intermediate hydroxide species [Pd(P^C^N)(OH)] showed an unexpected *N*-detachment, followed by room temperature rollover C–H activation on the pyrazole ring, which converts the formally anionic P^C^N pincer ligand into a formally dianionic P^C^C rollover-pincer ligand, finally affording the dinuclear species **100**. In contrast, with the methylated analogue PCN-Me, no rollover reactivity was observed and the mononuclear complex **101** was isolated and characterized (Figure 51) [28].

A DFT computational investigation revealed low energy barriers for pyrazole C–H activation following a σ-bond metathesis pathway, in line with the experimental data.

**Figure 52:**
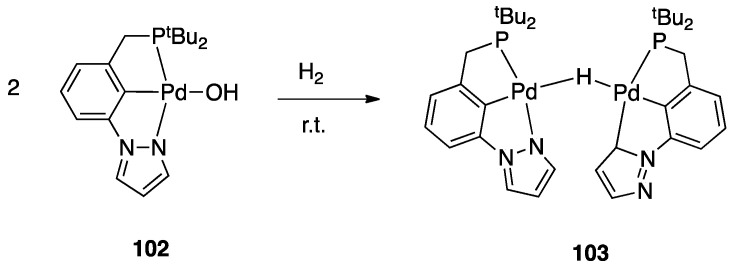
Synthesis of the dinuclear hydrido complex **103**. Adapted from *Chem. Eur. J.* **2019**, 9920–9929.

A few years later, the same group studied the hydrogenolysis of mono- and dinuclear Pd(II) hydroxides of the same ligands, **97** and **98**. Whereas reaction of the dinuclear hydroxo complexes [(P^C^N)_2_Pd_2_(µ-OH)]^+^ with H_2_ afforded the corresponding dinuclear hydrides [(P^C^N)_2_Pd_2_(m-H)]^+^, the same reaction of the mononuclear complex (PCN)Pd-OH (**102**) resulted in a rollover activation of one pyrazole ring, affording the dinuclear species [(PCNH)Pd](µ-H)[(PCC)Pd], **103**, bearing two different pincer ligands (P^C^N and P^C^C, Figure 52). The analogous reaction, under the same conditions, with the terminal hydroxo complex of the rollover-protected methylated ligand, **101**, did not produce any hydride. Reaction mechanisms for the hydrogenolysis of the monomeric and dimeric hydroxo species were proposed on the basis of DFT calculations [101].

**Figure 53:**
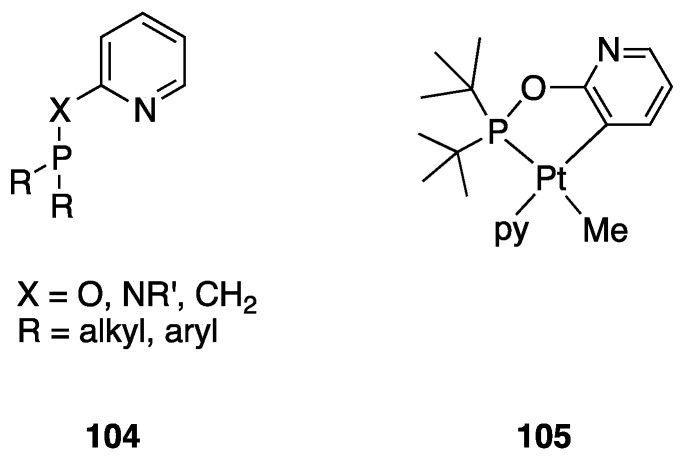
Rollover with P^N hemilabile ligands.

Another subclass of rollover cyclometalation involves P^N hemilabile ligands, such as phosphino pyridines. Goldberg and coworkers investigated the thermolysis behavior of two Pt(II) methyl-complexes [Pt(P^N)Me_2_] where P^N is (di-*tert*-butylphosphinito)pyridine or (di-*tert*-butylphosphino)-2-aminopyridine **104** (Figure 53). 

The authors started, as usual for rollover Pt(II) processes, from the dimethyl adduct [Pt(P^N)Me_2_] (P^N = **104**, X = O). Heating the complex in benzene, in the presence of pyridine, activated the C(3)-H pyridine bond by means of a rollover pathway, resulting in the synthesis of complex **105**. In contrast to the phosphinito complex, thermolysis of the adduct [Pt(P^N)Me_2_] (P^N = **104**, X = NH) resulted in a competition between intramolecular C–H activation via rollover cyclometalation and intermolecular benzene C–H activation, with formation of a mixture of CH_4_ and CH3D [102].

**Figure 54:**
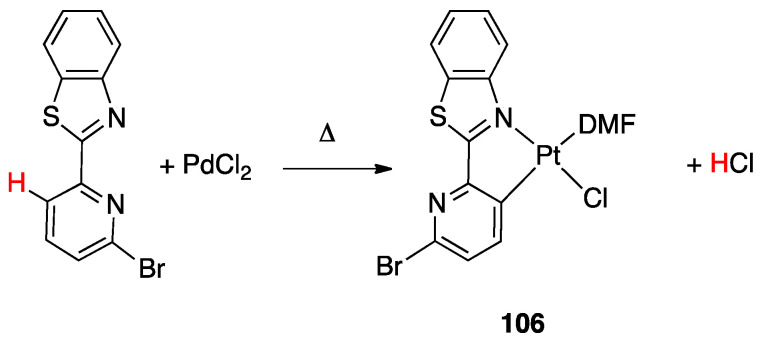
Rollover cylometalation with pyridinebenzothiazole. Adapted from *Chemistry Select* **2018**, *3*, 4133–4139.

Finally, one further ligand potentially able to coordinate in different ways, pyridinebenzothiazole, reacted with PdCl_2_ in DMF under mild heating (60 °C), activated the pyridine C(3)–H bond to afford the rollover complex **106**. Notably, the DMF solvent seems to play a key role in the rollover process, because use of other solvents resulted only in classical coordination (Figure 54) [103].

#### 4.2.2. Iridium and Rhodium Complexes

Iridium, as well as platinum, is able to produce the just discussed subclasses “carbene C^C rollover” and “pincer-rollover “complexes.

**Figure 55:**
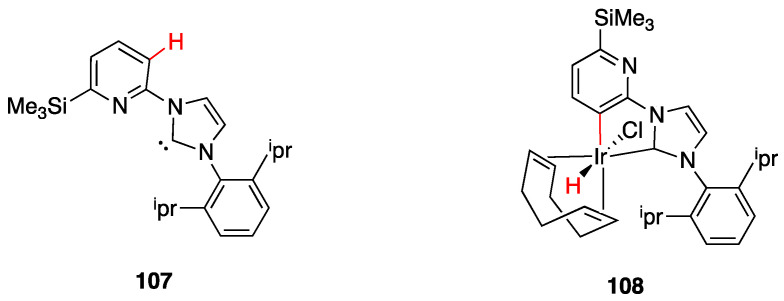
NHC-Ir(III) unusual C^C rollover complex **108**. Reproduced (redrawn) from Ref. [104] with permission from The Royal Society of Chemistry.

In 2002, the NHC-pyridine ligand **107**, 1-[(2-(6-trimethylsilyl)pyridyl]-3-[(2,6-di-iso-propyl)phenyl]imidazole-2-ylidene (Figure 55), showed for the first time room-temperature rollover C–H activation in a pyridine functionalized with a heterocyclic carbene, by reaction with the Ir(I) precursor Ir(η^4^-COD)Cl]_2_. This reaction allowed the isolation of the unusual Ir(III) C^C rollover complex **108** in quantitative yield. The reaction with the analogous Rh(I) complex [Rh(h^4^-COD)Cl]_2_ gave only the classical Rh(I) cyclometalated carbene complex [Rh(N^C)(h^4^-COD)]+ [104].

Years later, a second case of rollover cyclometalation promoted by [IrCp*Cl_2_]_2_ was reported with a bidentate triazolinylidene-pyrazole ligand. The reactivity of the NHC-rollover resulting C^C chelated complex was subsequently investigated in relation to insertion reactions [105].

**Figure 56:**
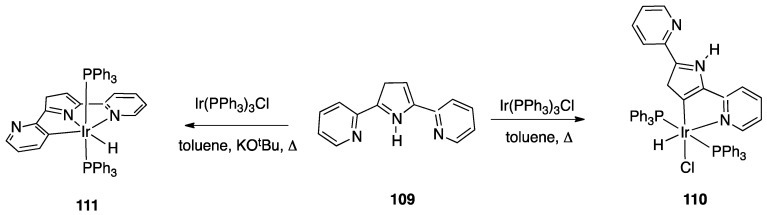
Rollover cyclometalation at the internal and external rings of 2-[5-(pyridin-2-yl)-1*H*-pyrrol-2-yl]pyridine. Adapted with permission from *Inorg. Chem*. **2020**, *59*, 2, 960–963. Copyright (2020) American Chemical Society.

The ligand 2-[5-(pyridin-2-yl)-1*H*-pyrrol-2-yl]pyridine, **109** (Figure 56), has a rich coordination ability, being able to coordinate in different ways, beyond the classical N^N^N tridentate pincer mode. According to reaction conditions, N^N, N^N^C (rollover activation of external pyridine ring), N^C (rollover activation of internal pyrrole) complexes were obtained by reaction with [Ir(PPh_3_)_3_Cl]. In particular, the Ir(III) compounds **110** and **111** were both formed in refluxing toluene, following two different C–H bond rollover activation pathways, in the pyrrole and pyridine rings, respectively. This case represents a very rare case of regioselective rollover activation between two different rollover pathways [106].

Examples of pseudo-rollover pathway, mediated both by Zr and Hf complexes, were shown by neutral Zr(IV) and Hf(IV) alkyl/amido complexes stabilized by a tridentate N^N^N ligand, which contains a “rolling” heterodentate benzoimidazole fragment. We only shortly mention this case because no metal-carbon bonds are formed, but the “rolling” benzoimidazole fragment gave different N^N^N tridentate complexes [107].

Three further examples regarding polypyrazolyl ligands occurred with ruthenium, nickel, and iron complexes.

The Ru(II) polypyrazolyl complex [(C(pz)_4_)Ru(P-(OCH_2_)_3_CEt)(CH_3_CN)Me][BAr′_4_], heated in a deuterated benzene solution in the presence of CH_3_CN underwent rollover C–H activation in one pyrazolyl ring to yield [(κ^3^-(N^C^N)C(pz)_4_)Ru(P(OCH_2_)_3_CEt)(CH_3_CN)2][BAr′_4_] and methane [108].

Intramolecular rollover C–H functionalization in a terdentate Ni azido complex Ni(N^N^N)(N_3_) occurred on one external pyrazolyl ring of the tridentate ligand, yielding an N^N^N^C tetradentate rollover complex [109].

Finally, a low-coordinate iron(II) complex with an N^N^N pincer ligand containing two hemilabile pyrazole groups underwent rollover C–H activation on one pyrazole affording an unusual pyrazolide-bridged iron(II) complex [110].

## 5. Organic Synthesis (Stochiometric Functionalization)

The rollover reaction allows activation at C(3) position in 2,2′-bipyridines as well as in analogous heteroaromatic ligands, a site which is typically hard to activate because these chelated ligands are strongly bonded to the metal and C–H bonds activation in this position requires partial ligand detachment.

Once regiospecifically activated by means of rollover metalation, the C–H bond functionalization becomes accessible. In this chapter, we report stoichiometric functionalization reactions, leaving catalytic processes to Section 6.

### 5.1. Palladium-Mediated Functionalization

**Figure 57:**
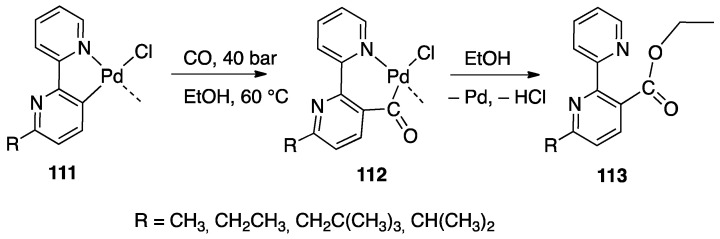
Synthesis of 2-(2-pyridin-2-yl)-6-alkyl-nicotinic esters by means of rollover cyclometalation. Reprinted from *J. Organomet. Chem*., **2010**, *695*, 256–259. Petretto, G.L.; Zucca, A.; Stoccoro S.; Cinellu, M.A.; Minghetti, G. Step by step Palladium mediated syntheses of new 2-(pyridin-2-yl)-6-R-nicotinic acids and esters. Copyright (2010), with permission from Elsevier.

Taking advantage of rollover cyclometalation, in 2010, Zucca and coworkers reported a first application of rollover functionalization of 2,2′-bipyridines. The authors showed that dinuclear palladium(II) rollover derivatives [Pd(N^C)Cl]_2_ **111** react in ethanol with CO, under pressure at 60 °C, affording the corresponding esters and acids, **112** and **113**, respectively (Figure 57) [111]. As key steps of the process were proposed insertion of CO into the palladium–carbon bond (leading an unstable six-membered acyl complex, subsequent nucleophilic attack of ethanol on the acylic carbonyl, and final extrusion of the organic product with reductive elimination of palladium. This reaction protocol allowed the synthesis of 2-(2-pyridin-2-yl)-6-alkyl-nicotinic acids or esters, derivatives which are rather rare and quite unreported in the case of the 6-substituted pyridine rings.

### 5.2. Platinum-Mediated Functionalization

**Figure 58:**
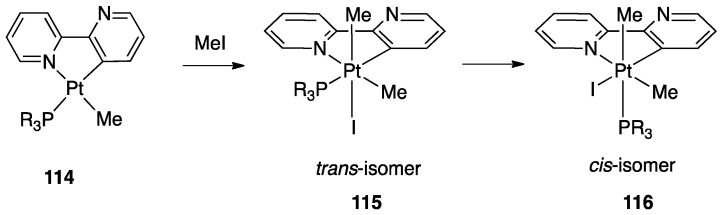
Oxidative addition reaction of MeI to Pt(II) rollover complexes.

Starting from Pt(II) rollover precursors, a sequence of oxidative addition-reductive elimination reactions can be useful for C–H bond functionalization of 2,2′-bipyridines. By reaction of Pt(II) rollover complexes [Pt(N^C)Me(L)] **114** (L = P donor ligands) with MeI, a series of analogous Pt(IV) complexes are readily accessible (Figure 58).

This oxidative addition reaction has been studied in detail by several groups; as for classical cycloplatinated complexes, the reaction of rollover analogues with MeI proceeds thorough the classical S_N_2 oxidative addition mechanism. The reactions afford the analogous Pt(IV) complexes [Pt(NC)(L)(Me)_2_I]; even though several isomers can be formed, due to *trans*-phobia, only fac PtC_3_ isomers are expected to be fairly stable, so that, considering the presence of the cyclometalated ligand, only two isomers should realistically be formed, i) the “*trans*” isomer **115**, which is expected to be the kinetic product, with the incoming ligands CH_3_ and I in mutual *trans* position, and the phosphane in “equatorial” position with respect to the bipyridine plane); ii) the “*cis*” isomer **116**, i.e., the thermodynamic product, with the phosphane ligand in “axial” position.

Kinetic and thermodynamic factors of the oxidative-addition reaction are regulated by a mix of electronic and steric factors: electron-richer Pt(II) complexes will react faster, so that PMe_3_ complexes will be advantaged towards PPh_3_ analogues. At variance, the PMe_3_ ligand, due to its smaller cone angle, will remain longer in its position, and the kinetic isomer (PR_3_ in “equatorial positions) can be isolated and characterized, even by X-ray spectroscopy [112]. With bulkier phosphanes, such as PPh_3_ or PCy_3_, the isomerization to the thermodynamic product, i.e., the *cis* product, is faster.

The influence of the cyclometalated ligand was studied by Rashidi and coworkers who reported the reactivity of the roll-over platinum(II) complexes [PtMe(bpy-*H*)(PPh_2_Me)] and [PtMe(bpy-*H*)(PPh_3_)], **114**, with MeI, to give the corresponding rollover Pt(IV) complexes [PtMe_2_(bpy-*H*)(PR_3_)I]. Kinetic data suggested a classical S_N_2 mechanism with large negative DS^‡^ values. The PPh_3_ complex reacted slower with MeI than the PPh_2_Me one likely due to the stronger donor ability and the less steric hindrance of the PPh_2_Me ligand. Comparison with the classical cyclometalated complexes [PtMe(ppy-*H*)(L)], **117**, (ppy-*H* = 2-phenylpyridinate, L = PPh_3_, PPh_2_Me) showed that rollover complexes reacted slower than phenylpyridine analog. This was attributed to the presence of the uncoordinated nitrogen atom which makes rollover coordinated 2,2′-bpy a slightly weaker donor than cyclometalated 2-phenylpyridine [113].

**Figure 59:**
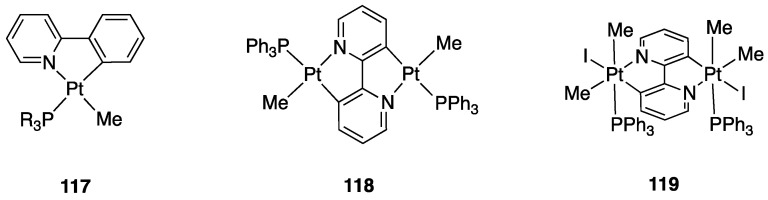
Mono- and di-nuclear complexes **117**–**119**.

The same reaction was studied with the corresponding dinuclear double rollover complex **118**, [Pt_2_(m-bpy-2*H*)Me_2_(PPh_3_)_2_]: Additionally, in this case, oxidative addition occurs, to give the dinuclear Pt(IV) complex **119** (Figure 59). The rate for the oxidative addition reaction was found to be almost 3–5 times slower in the second step (i.e., that on the second platinum center) as compared to the first one. This result is important, because it confirms transmission of electronic effects through the delocalized rollover bipy ligand. In comparison with the corresponding monomeric [Pt(bipy-*H*)Me(PPh_3_)] complex, in the dinuclear species **118**, the rate for the oxidative addition reaction was found to be higher in step 1 and lower in step 2 [114].

**Figure 60:**
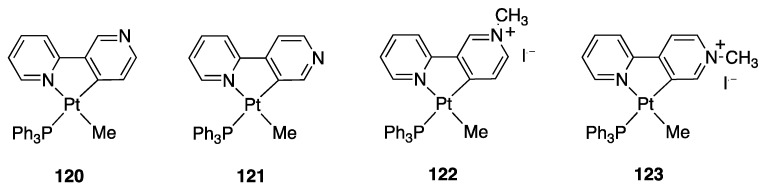
Cyclometalated complexes derived from bipyridine isomers.

The Pt(II) rollover complexes of these series have several nucleophilic centers, in particular, the uncoordinated nitrogen and the platinum(II). Hosseini and coworkers studied the reaction of three cyclometalated isomers [Pt(N^C)Me(PPh_3_)], **114**, **120**, and **121**, with methyl iodide finding that only in the rollover complex, the stronger nucleophile is the platinum center, affording the Pt(IV) complex **116**. In contrast, complexes **120** and **121** were predicted to react, by DFT calculations, with the nitrogen donor, to form *N*-methylated cyclometalated complexes **122** and **123** (Figure 60). The reasons for this different behavior in selectivity were found in the energy barrier needed for *N*-methylation (higher in the rollover complex) vs. oxidative-addition reactions [115].

**Figure 61:**
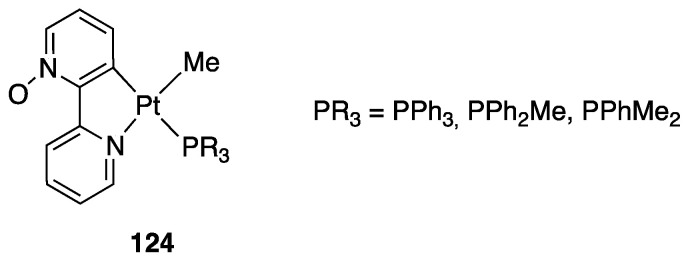
Pt(II) N-O bipyridine-derived rollover complexes **124**.

Shahsavari and coworkers studied the same oxidative reaction with the Pt(II) N-O bipyridine-derived rollover complexes **124**, comparing three phosphane ligands: PPh_3_, PPh_2_Me, and PPhMe_2_ (Figure 61). The kinetic results showed that the electronic effects of the phosphanes determine the reaction rates with, as expected, the following trend: PPhMe_2_ > PPh_2_Me > PPh_3_. As previously found, the *trans*-*cis* isomerization (kinetic to thermodynamic product) is mostly affected by steric effects due to phosphane ligands, so that the kinetic trend is PPh_3_ > PPh_2_Me > PPhMe_2_ [116].

The same reaction, with deuterated CD_3_I, was monitored by NMR, evidencing a scrambling between the incoming CD_3_ ligand and the preexisting CH_3_ ligand, which exchange their mutual positions.

In a successive paper, the same group studied the influence of the rollover cyclometalated ligand, comparing the cyclometalated N-O bipyridyl ligand with the classical deprotonated phenylpyridine and benzo[h]quinoline ligands [117]. The study showed that the reaction rate in the oxidative addition of MeI is significantly slower with the bipy-*N*-oxide ligands, showing the effect of the N-O group on the Pt(II) reactivity.

**Figure 62:**
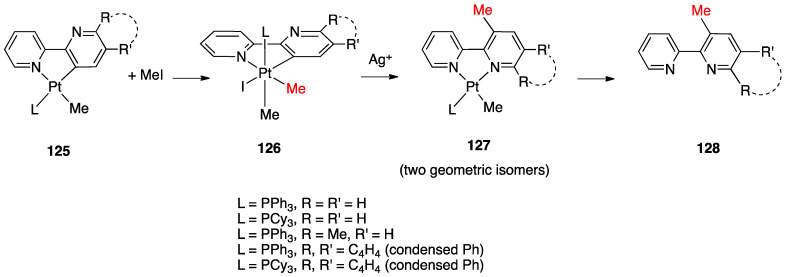
Pt(II)-Pt(IV) mediated functionalization of 2,2′-bipyridines by means of rollover C–H bond activation. Adapted with permission from *Chem. A Eur. J.* **2014**, *20*, 5501–5510. Copyright (2014) John Wiley & Sons, Inc.

The oxidative addition reaction can be coupled with a successive reductive elimination step with C-C coupling in order to obtain a functionalized bipyridine. Starting from the Pt(II) rollover complexes **125** (Figure 62), reaction with MeI gave the corresponding Pt(IV) complex **126**. A subsequent reductive elimination promoted by abstraction of the iodide ligand resulted in C(sp^2^)-C(sp^3^) bond formation to finally afford the 3-functionalized 2,2′-bipyridines **128**: 3-methyl-2,2′-bipyridine, 3,6-dimethyl-2,2′-bipyridine, as well as 3-methyl-2-(2′-pyridyl)-quinoline, which were isolated and characterized [118].

The reductive elimination step involves a rare C(sp^3^)-C(sp^2^) coupling, in place of the more common C(sp^3^)-C(sp^3^) one (in this case, with ethane elimination), and whether this reductive elimination takes place or not seems to be governed principally by steric factors, as indicated by the behavior of PPh_3_, PMe_3_, and PCy_3_ complexes. Worth to note, the reaction occurs only in the presence of the uncoordinated nitrogen, typical of rollover complexes, because it takes advantage of a retro-rollover pathways, to give the intermediate N^N chelated analogues **127**. For this reason, the reaction is unique to rollover complexes; indeed, the analogous phenylpyridine cyclometalated complexes do not exhibit C–C coupling when treated with silver salts.

This route constitutes a new stoichiometric method for the activation and functionalization of C(3)-H position in bidentate heterocyclic compounds.

### 5.3. Ruthenium-Mediated Functionalization

**Figure 63:**
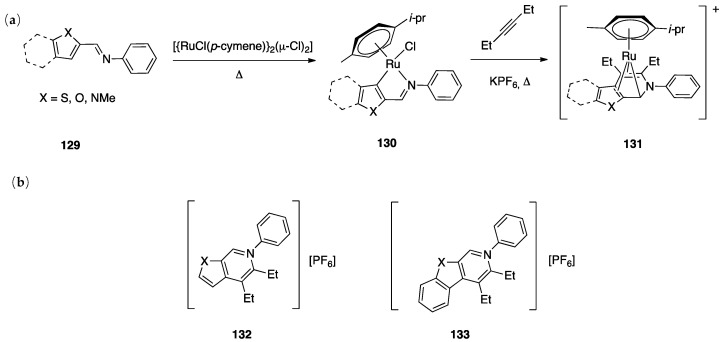
(**a**) C-C coupling mediated by ruthenium rollover cyclometalation; (**b**) fused bis-heterocycles **132** and **133.** Adapted with permission from *Chem. A Eur. J*. **2012**, *18*, 15178–15189. Copyright (2012) John Wiley & Sons, Inc.

A third case of rollover metal-mediated C–H bond functionalization involves a series of ruthenium complexes with cyclometalated imines-based heterocycles [119]. The reaction of [{RuCl(h^6^-*p*-cymene)}(m-Cl)_2_] with a series of imines-based heterocyclic ligands (**129**) in the presence of Cu(OAc)_2_ (Figure 63) resulted in the rollover cyclometalation due to C–H bond activation in the C(3) position of the five-membered heterocyclic ring (thiophene, benzothiophene, furan, benzofuran, pyrrole, and indole derivatives) affording rollover complexes **130**. These complexes unexpectedly react with 3-hexyne to give complexes **131.**

Successive reaction with CuCl_2_, promotes rearomatization of the ligand and release of the corresponding fused bis-heterocycles **132** and **133** (X = S, O, NMe) providing a novel synthetic method for the preparation of this family of compounds.

Furthermore, the authors showed that it is also possible to obtain these organic products in a one-pot sequential procedure allowing the synthesis of a series of new derivatives.

**Figure 64:**
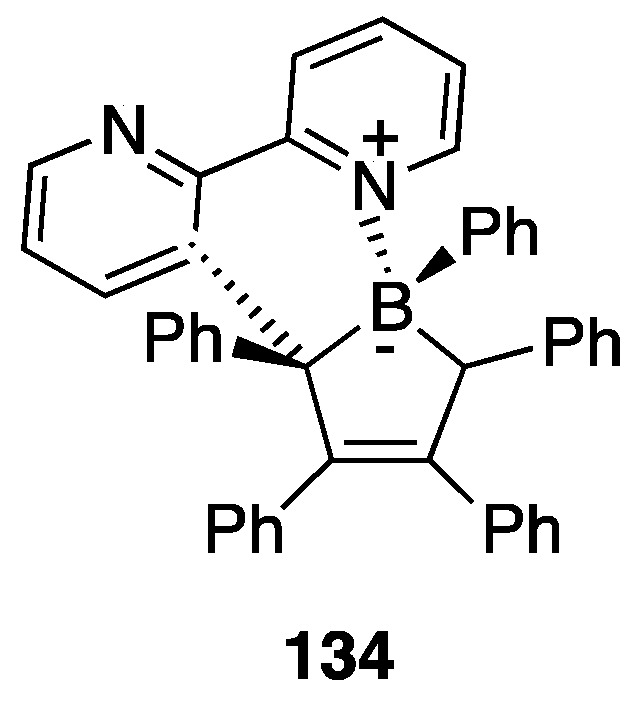
Reproduced (redrawn) from Ref. [120] with permission from The Royal Society of Chemistry.

Finally, in may be mentioned a particular case of C(3) bond functionalization in 2,2′-bipyridine which does not involve transition metal coordination. The reaction of pentaphenylborole with 2,2′-bipyridine forms an adduct which, upon thermolysis, converts to the *ortho* addition product **134** (Figure 64).

An analogous reaction occurs with 2-phenylpyridine. Such reactivity is uncommon for main group elements and provides application to access boron compounds [120].

## 6. Catalysis

Cyclometalated compounds have been widely used as catalysts [121,122]. As an example, pincer complexes have demonstrated to be active in large series of catalytic reactions, such as C–C bond coupling, transfer hydrogenation, alkane dehydrogenation, and polymerization [123].

Among the various electronic and steric properties furnished by cyclometalated ligands, it is worth to remind that cyclometalation generates a formal carbanionic ligand, that enhances the electron density on the metal center. In addition to the classical case, rollover cyclometalation is able to act as a reversible process, allowing to play an uncommon role in catalytic reactions.

This role can assume two different aspects: (i) rollover cyclometalated complexes can be used as catalytic precursors, or (ii) the rollover process can be one step of a catalytic cycle. In this case, the catalytic precursor may be a non-organometallic species, and rollover complexes are formed, react, and decoordinate in the course of the catalytic cycle.

### 6.1. Rollover Complexes as Catalysts

**Figure 65:**
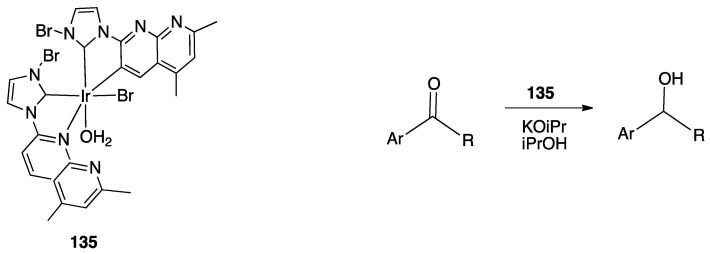
Catalytic activity of the Ir-carbene-rollover complex 135. Adapted with permission from *Inorg. Chem.* **2009**, *48*, 23, 11114–11122. Copyright (2009) American Chemical Society.

To the best of our knowledge, the first catalytic application of a rollover complex has been reported in 2009. Bera and coworkers synthesized the uncommon Ir(III) complex **135** (Figure 65) which incorporates a cyclometalated *N*-heterocyclic carbene and a rare “carbene-rollover” ligand. The complex, characterized by means of X-ray spectroscopy, proved to be catalytically active in the hydrogen transfer reaction of aromatic and heteroaromatic ketones at room temperature with high yields (Figure 65) [124].

#### 6.1.1. Platinum Catalyzed Reactions

**Figure 66:**
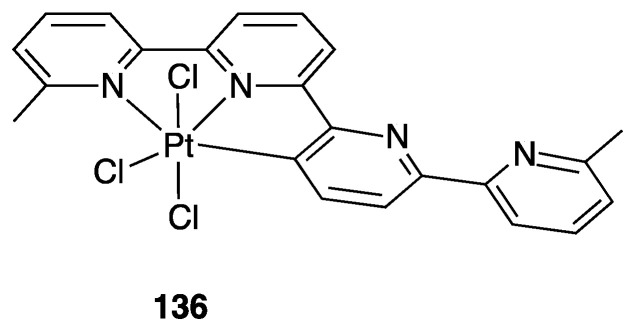
Pt(IV) rollover complex **136**.

A few years later, a new Pt(IV) rollover complex, [Pt(N^N^C)Cl_3_], **136** (Figure 66), was prepared by reaction of PtCl_2_ with a dimethyl quaterpyridine.

**Figure 67:**
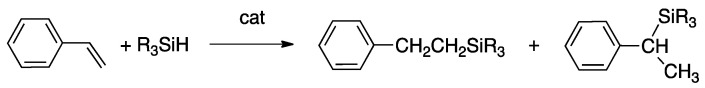
Hydrosilylation of styrene.

No experimental conditions for the synthesis were provided by the authors. The rollover complex **136**, isolated and characterized by means, inter alia, of X-ray crystallography, showed to be a highly selective catalyst precursor in the hydrosilylation of styrene and terminal alkynes (Figure 67) [125].

#### 6.1.2. Ruthenium Catalyzed Reactions

As seen in Section 4.1.7, Niedner-Schatteburg, Thiel, and coworkers presented in 2013 the Ru(II) complex **68**, able to convert into the corresponding rollover complex **69**.

Complex **68** was found to catalyze both the transfer hydrogenation of ketones and the reductive amination of aromatic aldehydes. The reactions proceed without presence of a base, an important feature which, for instance, prevents chiral compounds to undergo racemization. Indeed, the reported Ru(II)-rollover hydrogen transfer of arylalkyl ketones constitutes the first case of an air stable “phosphane-free” and “base free” hydrogen transfer catalysts [90]. The experimental and DFT data confirm that the key step of the self-activation of the catalyst is the C–H bond rollover activation in the pyrimidine ring.

Four years later, a detailed mechanistic study on the process, extended to other related ligands, allowed the classification of the ligands according to their stereoelectronic properties. Additionally, in this case, rollover activation of the bidentate nitrogen ligand was proposed to be a key step of the process. DFT calculations, CID EIS-MS (CID = collision induced dissociation) and X-ray structural data helped the authors to conclude that a mix of electronic and steric factors were active in weakening critical metal-ligand bonds, favoring roll-over cyclometalation. In particular, the higher activity was shown with a tertiary amino substituent in the pyrimidine ring.

**Figure 68:**
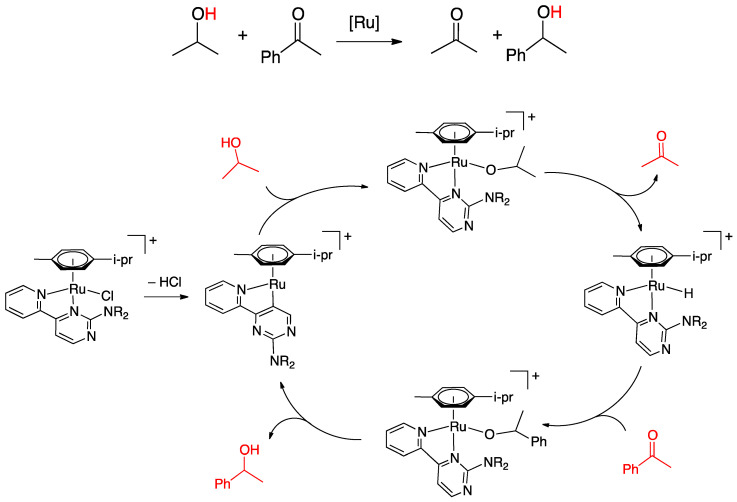
Proposed mechanism for catalytic transfer hydrogenation in the absence of an external base. Reprinted from *J.* *Organomet. Chem*. **2018**, *863*, 30–43. Leist, M.; Kerner, C.; Ghoochany, L.T.; Farsadpour, S.; Fizia, A.; Neu, J.P.; Schön, F.; Sun, Y.; Oelkers, B.; Lang, J.; Menges, F.; Niedner-Schatteburg, G.; Salih, K.S.M.; R. Thiel, W.R. Roll-over cyclometalation: A versatile tool to enhance the catalytic activity of transition metal complexes. Copyright (2018), with permission from Elsevier.

A proposed catalytic cycle (Figure 68), involving hydrogen transfer reactions, rollover and retro-rollover reactions, was supported by NMR spectroscopy, kinetic studies, IR-MPD, ESI-MS, and DFT calculations [18,71,72,126].

Developing the potentialities and applicability of the Ru(II)-rollover catalyst, Thiel and co-workers also showed that complex **69** is highly active in catalyzing the reductive amination of aromatic aldehydes with imines in 2-propanol, which acts both as the hydrogen source and solvent [127].

The hydrogenation of carbon monoxide to formate is catalyzed by another Ru(II) complex, containing a pincer N^C^N di-pyridyl-imidazol-2-ylidene ligand [128]. A key step of the catalytic process was proposed to be a rollover switch of a pyridine ligand in a bidentate former-pincer complex, to afford an unusual C(carbene)^C(pyridyl) chelate. The process allows hydride exchange likely occurring via a dihydrogen complex according to the Perutz and Sabo-Etienne CAM mechanism (complex assisted metathesis) [129].

#### 6.1.3. Palladium-Catalyzed Reactions

**Figure 69:**
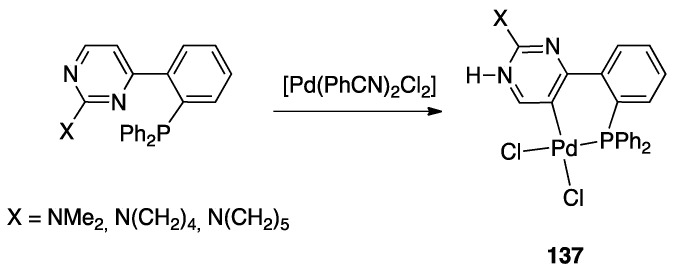
Pd(II) rollover complexes with (2-aminopyrimidinyl) phosphanes. Adapted from *Eur. J. Inorg. Chem*. **2011**, 4603–4609.

Thiel and coworkers synthesized a series of Pd(II) complexes with (2-aminopyrimidinyl)phosphanes. Among the complexes, besides [Pd(N^N)Cl_2_] adducts, the rollover complexes [Pd(P^C)Cl_2_], **137**, were isolated and studied. In these complexes, the ligand, due to nitrogen protonation, acts as a formally neutral ligand and may be regarded as a mesoionic NHC ligand. The new complexes, adducts, and rollover cyclometalates were investigated as catalysts in the Suzuki–Miyaura coupling reaction of aryl halides with phenylboronic acid at room temperature (Figure 69). The experimental data showed that slight changes at the amino group of the ligands result in pronounced differences both in the stability and catalytic activity of the corresponding complexes [130].

The same group, years later, reported the elevated catalytic activities of the uncommon rollover NHC complex **89** (described in Section 4.2.1) in Suzuki–Miyaura cross-coupling reactions of arylboronic acids with aryl chlorides [100].

### 6.2. Rollover Pathways in Catalytic Cycles

**Figure 70:**
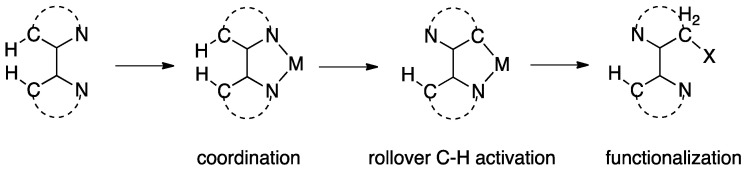
Rollover activation and functionalization strategy.

Several aspects of the rollover reaction, such as its reversibility, make it useful for catalytic applications. A functionalization strategy for C–C coupling reactions may involve coordination of a bidentate ligand, rollover C–H bond activation, metal-mediated functionalization, and final decoordination of the functionalized ligand. Other applications may involve a rollover-retro-rollover sequence with hydrogen transfer reactions (Figure 70).

In the course of the last years, several synthetic catalytic protocols involving rollover C–H activation and functionalization have been reported. We will present the results according to the metal used to catalyze the reactions.

#### 6.2.1. Rhodium

##### C–C Couplings

**Figure 71:**
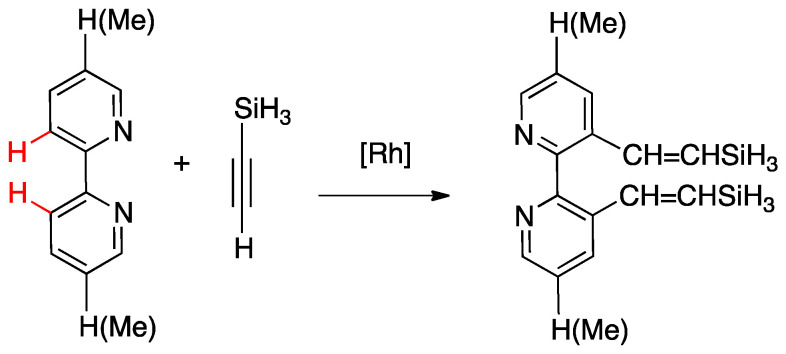
Rh-catalyzed double C–H bond rollover functionalization in 2,2′-bipyridine.

Being relatively rare, at least until a few years ago, rollover cyclometalation and/or activation was not always recognized. To the best of our knowledge, the first case of the rollover catalyzed process was reported in 2009 by Miura et al., which showed that [RhCl(COD]_2_ catalyzes a consecutive double C–H bond activation in 2,2′-bipyridine affording the corresponding 3,3′-dialkenylated product by reaction with silylacetylene (Figure 71) [131].

In the following years, the Chang’s group developed a catalytic method for the functionalization of pyridine-based bi- and tri-dentate ligands by means of a rollover pathway. The first paper of the series, in 2012 [132], reported the hydroarylation of alkenes and alkynes with 2,2′-bipyridines and 2,2′-biquinolines promoted by Rh(III)-NHC (Nitrogen Heterocyclic carbene) catalysts.

**Figure 72:**
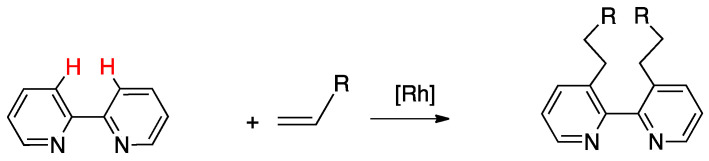
Rollover hydroarylation of alkenes. Adapted with permission from *J. Am. Chem. Soc.* **2012**, *134*, 42, 17778−17788. Copyright (2012) American Chemical Society.

Two subsequent rollover cyclometalations give rise to double functionalization of the bidentate molecules (Figure 72). The rollover pathway is facilitated by the strong *trans* effect of the coordinated *N*-heterocyclic carbenes, which labilize the rhodium–pyridyl bond in *trans* position. The method enables a highly selective and efficient double functionalization of 2,2′-bipyridines and 2,2′-biquinolines.

Application of the same protocol to tridentate N^N^N ligands, such as 2,2′:6′,2″-terpyridine or related ligands, resulted in regioselective bis-alkylation of the terdentate ligands.

The regioselectivity in the double C–H functionalization can be ascribed to a bis-rollover pathway preferring, as previously seen with Pt(II) [25], the central pyridine ring. The method allows the preparation, with high yields, of a series of functionalized tridentate compounds, with application to a wide range of tridentate heteroarenes and alkene reactants [133].

**Figure 73:**
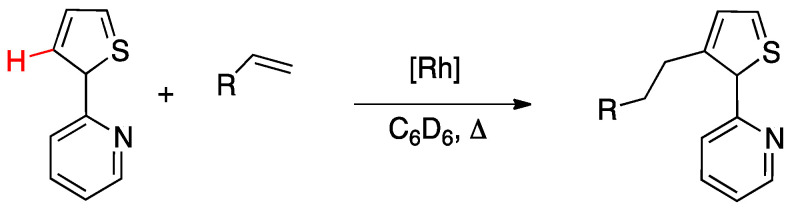
Rh-catalyzed selective rollover functionalization of 2-(2-thienyl)pyridine.

Rh(III)-NHC complexes (NHC = 1,3-bis-(2,6-diisopropylphenyl)imidazol-2-ylidene) were also found to catalyze the selective rollover functionalization of 2-(2-thienyl)pyridine with a series of alkenes and internal alkynes. In course of the reaction, a rollover C–H bond activation converts the N^S chelated intermediate into a C^N rollover complex, by means of a proposed oxidative-addition process (Figure 73) [25].

As we will see hereinafter, the [RhCp*Cl_2_]_2_ catalyst has been used for a large series of rollover-catalyzed reaction. Recently, the regioselective C–H bond functionalization on tridentate 2,2′-bipyridine-6-carboxamides by means of a rollover pathway was reported [134].

**Figure 74:**
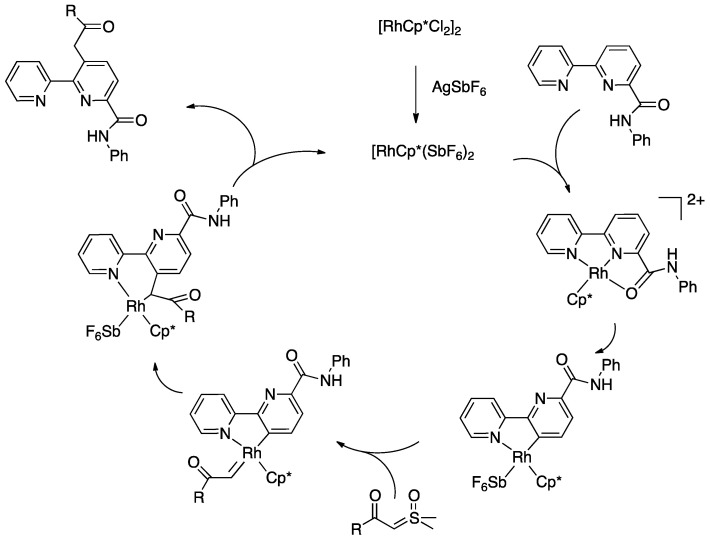
Proposed catalytic cycle for the alkylation of 2,2′-bipyridine-6-carboxamides. Adapted with permission from *Org. Lett.* **2019**, *21*, 6366–6369. Copyright (2019) American Chemical Society.

The authors showed that a series of 2,2′-bipyridine-6-carboxamides can be selectively monoalkylated at the C(3) position. In the proposed catalytic cycle, reported in Figure 74, after coordination of the tridentate ligand and rollover cyclometalation, reaction with sulfoxonium ylides gives carbene complexes. Subsequently, migratory insertion of the coordinated carbene into the Rh-C(3) bond is followed by protonolysis, to give the 3-alkylated products in high yields. This reaction protocol has a large applicability (the authors furnish 40 examples) and also showed an excellent tolerance to functional groups.

In contrast to this “internal pyridine ring activation”, the Cheng group, working with the same ligands, presented a Pd(II)-catalyzed process in which the rollover process activates only the external pyridine ring (see later).

##### Cycloannulation Reactions

**Figure 75:**
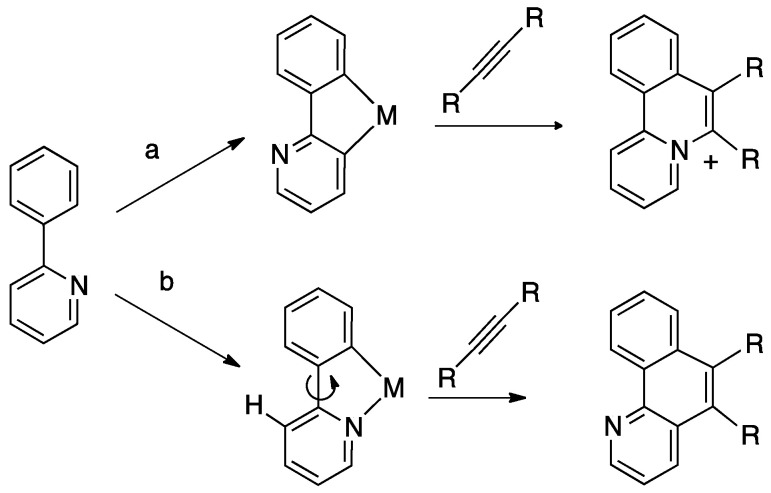
Possible cycloannulation pathways with single and double C–H bond activations. Adapted with permission from *Org. Lett.* **2015**, *17*, 12, 3130–3133. Copyright (2015) American Chemical Society.

Fused polycyclic heteroarenes constitute an important scaffold in organic chemistry. In this context, it is well-known the rhodium(III)-catalyzed annulation of 2-phenylpyridines with alkynes: the reaction proceeds through successive C–C and C–N bond formations and is initiated by a C–H bond activation through cyclometalation [135,136,137]. In this framework, the [RhCp*Cl_2_]_2_ catalyst has been often successfully used. A different series of annulation reactions involves a twofold C–H bond cleavage and C–C bond formation and is initiated by means of rollover C–H bond activation and metalation. This second case appears to be attractive because it enables the assembly of different polycyclic systems. The two possible annulation reactions are reported in Figure 75. Path a represents single C–H bond cleavage and C–C and C–N bond formations, whereas rollover path b represents double C–H bond cleavage and twofold C–C bond formation.

Although the second pathway of 4 + 2 cycloannulation looks attractive, to the best of our knowledge, it has not been observed yet with 2-phenylpyridine. In contrast, several other heteroaromatic nitrogen ligands have been functionalized taking advantage of this reaction.

**Figure 76:**
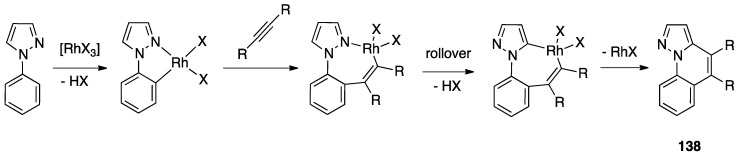
Cycloannulation reaction of 1-phenyl-pyrazoles alkynes. Adapted with permission from. *J. Org. Chem.* **2011**, *76*, 1, 13–24. Copyright (2011) American Chemical Society.

Firstly, in 2011, Miura and coworkers reported the oxidative coupling of phenylazoles with internal alkynes, in the presence of [RhCp*Cl_2_]_2_ as catalyst and Cu(OAc)_2_ as oxidant. Several products were formed by means of double or even quadruple C–H bond activation. The reaction pathway was found to be highly dependent on the reaction conditions employed, and activation preferentially took place at the electron deficient sites in the aromatic rings. Among the various reaction pathways and the ample series of substrates and products, it should be mentioned for the first time a rollover annulation pathway, regarding 1-phenyl-pyrazoles, which implies a two-fold C–H bond activation to give the annulated compounds **138** (Figure 76) [138].

**Figure 77:**
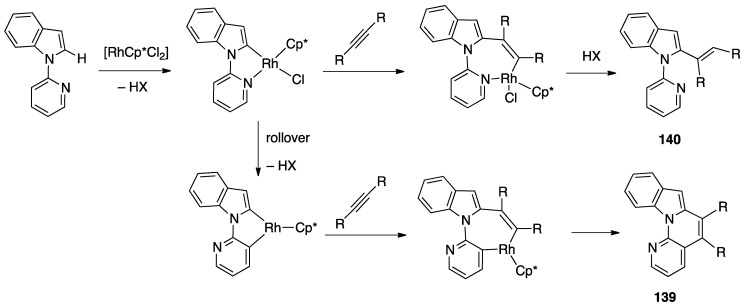
Plausible reaction mechanisms in the Rh-catalyzed dehydrogenative coupling of *N*-pyridylindoles with alkynes. Adapted with permission from *Org. Lett.* **2015**, *17*, 12, 3130–3133. Copyright (2015) American Chemical Society.

Modification of the heteroaromatic scaffold opens vast synthetic perspectives. Following their studies, the same research group reported the rhodium-catalyzed dehydrogenative coupling of *N*-pyridylindoles with alkynes, in the presence of copper or silver salts. The reaction easily afforded indolo [1,2-a]-naphthyridine **139** derivatives following a rollover pathway [139]. According to reaction conditions (Cu or Ag salt as oxidant, choice of solvent, temperature, substrate), in addition to compound **139**, also the C^2^-alkenylated product **140** was formed in different amounts. A plausible reaction mechanism, proposed by the authors, is reported in Figure 77.

This reaction protocol was successfully applied to a number of tetra-, penta-, and hexacyclic *N*-containing heteroaromatic compounds, showing to be of ample interest, also because the annulation products exhibit intense fluorescence in the solid state.

**Figure 78:**
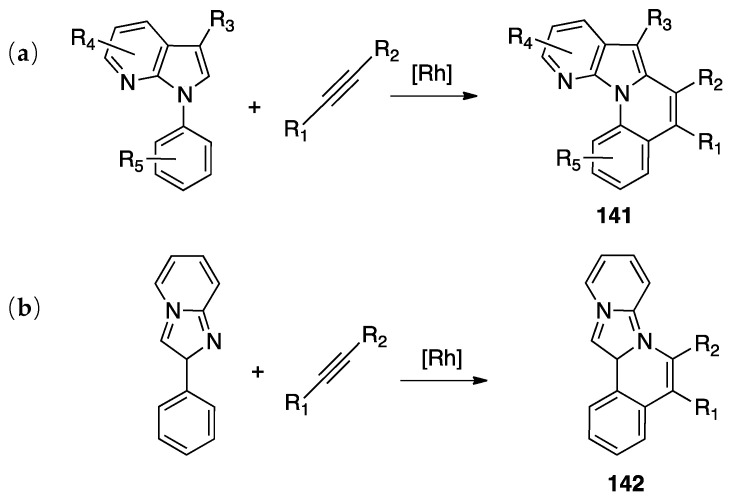
Synthesis of 5,6-disubstituted naphtho[1′,2′:4,5]imidazo[1,2-a]pyridines. (**a**) 7-azaindoles and alkynes; (**b**) 2-phenylimidazo[1,2-a]pyridines with alkynes. Adapted with permission from. *J. Org. Chem.* **2015**, *80*, 7, 3471–3479. Copyright (2015) American Chemical Society.

Other cases of oxidative rollover-annulation reactions catalyzed by Cp*Rh(III) complexes involve the oxidative annulation of 7-azaindoles and alkynes, taking advantage of the high reactivity of the 2-position in the pyrrole ring (Figure 78a) [140] and of 2-phenylimidazo[1,2-a]pyridines with alkynes, to give 5,6-disubstituted naphtho[1′,2′:4,5]imidazo[1,2-a]pyridines **141** and **142** (Figure 78b) [141].

**Figure 79:**
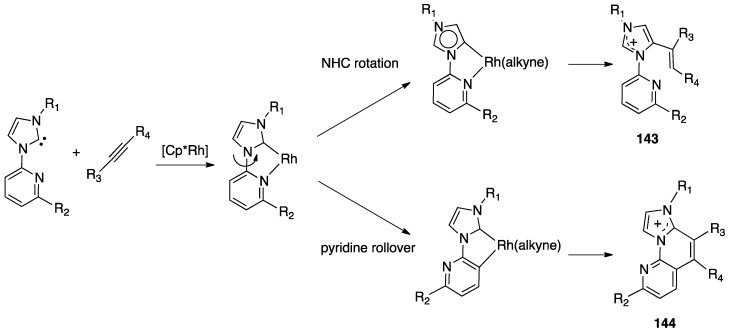
Bimodal rollover C–H bond activation: depending on steric (R1, R2) and electronic factors (R3, R4). Adapted with permission from *ACS Catal.* **2016**, *6*, 709–713 (https://pubs.acs.org/doi/10.1021/acscatal.5b02540). Copyright (2016) American Chemical Society. Further permissions related to the material excerpted should be directed to the ACS.

The above-described method was also successfully applied to the annulation of pyridine-imidazolium substrates. Choudhury and coworkers reacted both normal [142] and abnormal [143] CHN imidazolium-based carbenes with internal alkynes, finding how stereoelectronic factors drive the process. Different rollover C–H activation pathways were operative (NHC rotation vs pyridine rollover) depending on the properties of the organometallic intermediate, to give compounds **143** or **144** (Figure 79).

An important novelty of these studies is the “controlled mechanistic dichotomy”, which allowed to switch the pathway of the functionalization reaction. The authors found that the reactions were selectively driven toward rollover or non-rollover C–H functionalization routes on the basis of the steric and electronic properties of the rhodium(III) metallacyclic intermediates. In the presence of copper(II) acetate and non-polar solvent, the basic and coordinating acetate anion favor the rollover pathway leading to annulation products. In contrast, working in polar solvents with Cu(BF4)2 the influence of the BF4 anion (both weakly basic and coordinating) switches the reaction towards double alkyne insertion, affording C-N annulated cationic products [144].

Other cases of Cp*Rh(III)-catalyzed oxidative rollover-annulations with alkynes involve a series of pyridinones, to produce functionalized 4*H*-quinolizin-4-ones [145], and *o*-alkenyl anilides, from which unexpected naphthalene adducts were formed [146].

Recently, the cycloannulation Rh(III)Cp* catalytic protocol was extended to the functionalization of 2-(1*H*-pyrazol-1-yl)pyridine with internal alkynes. The reaction provided a switchable solvent-controlled C–H functionalization, affording alkenylated 2-(1*H*-pyrazol-1-yl)pyridine or indazole derivatives, with moderate to good yields. In both cases, only the pyrazolyl ring was involved in the C–H activation. Control experiments, conduced with “pre-rollover“ and “post-rollover” intermediates, suggested a rollover pathway for both cases [147].

The same strategy ([RhCp*Cl2]2 as catalyst, heating in the presence of AgOAc and a base) was adopted for 2,2′-bipyridine functionalization. Experimental and DFT data showed a considerable substituent effect: as often observed for substituted 2,2′-bipyridines, a substituent in position 6 plays an important role, weakening the nearby Rh–N bond and facilitating subsequent rollover C–H bond activation and functionalization [148].

Furthermore, the Rh-catalyzed oxidative annulation of imidazopyridines and indazoles was recently achieved using vinylene carbonate as a “vinylene transfer agent”. In the proposed reaction mechanism, a rollover C–H bond activation was a key step in the catalytic cycle [149].

The [RhCp*Cl_2_]_2_ complex was also reported to catalyze a divergent C–H bond activation and functionalization in aromatic picolinamide derivatives. Two sites for C–H activation are available, in pyridine and arene rings. Activation afforded isoquinoline derivatives or ortho-olefinated benzylamine (or phenethylamine) compounds, based on the mechanism involved. This different reactivity was achieved switching the catalyst between Rh(III) and Rh(I), which results in a modification of the mechanism of the process. In addition, a series of experimental and DFT mechanistic studies helped to understand insights of the divergent regiochemical outcome [150].

**Figure 80:**
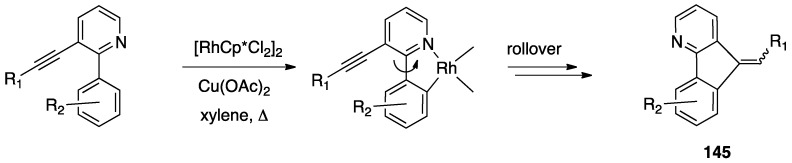
Internal activation reaction of 3-alkynyl arylpyridines. Adapted with permission from *Org. Lett.* **2012**, *14*, 19, 5106–5109. Copyright (2012) American Chemical Society.

Internal rotation of pyridine rings can assume various shapes: an internal rotation, without rollover C–H bond activation promotes intramolecular reaction of 3-alkynyl and 3-alkenyl-2-arylpyridines, in the presence of [Cp*RhCl_2_]_2_ as catalyst and Cu(OAc)_2_ as catalytic additive. The reaction allowed the synthesis of new carbon skeleton of 4-azafluorenes **145** (Figure 80) [151].

**Figure 81:**
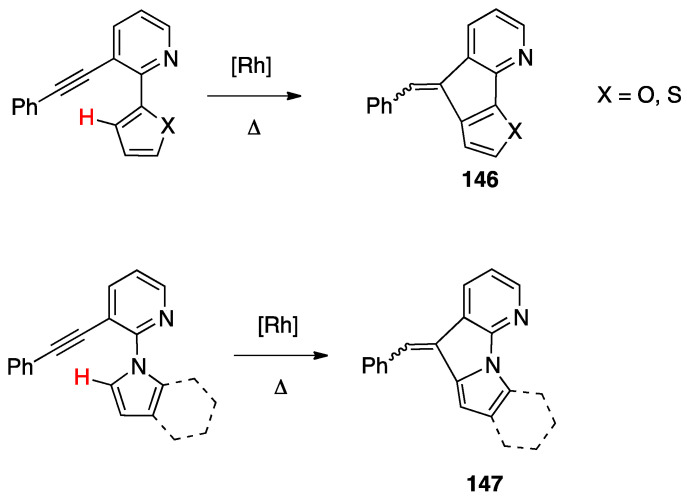
Intramolecular alkyne insertion into 3-Alkynyl-2-heteroarylpyridines. Redrawn from *Heteroatom Chemistry*, **2014**, *25,* 5*,* 379–388, Ref. [152].

Two years later, the Rh(III)-catalyzed rollover method was extended to the synthesis of a series of multicyclic heterocycles. The authors showed that intramolecular reaction of 3-alkynyl-2-heteroarylpyridines afforded tri- and tetracyclic nitrogen heterocycles **146** and **147** incorporating also oxygen, sulfur, or nitrogen (Figure 81). Catalytic amounts of pivalate or acetate salts were found to facilitate the reaction. Furthermore, the analogous reaction of 3-aryloyl-2-arylpyridines gave 7-substituted 4-azafluoren-9-ols. In order to prove the role of the pyridine nitrogen atom in the process, the authors showed that no reaction proceeded starting from nitrogen-free analogues, or positional isomers, not able to assist C–H bond cleavage and the subsequent rollover process [152].

Internal rotation of a phenyl ring in a cyclometalated 2-phenylpyridine also occurs in the course of catalytic studies on the reaction with [RhCp*Cl_2_]_2_ and cyclopropenones [153].

##### Rotation of a Phenyl Ring

**Figure 82:**
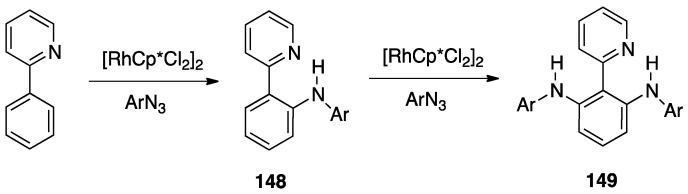
One example of rhodium-catalyzed mono and diamination of arenes. Adapted with permission from *Org. Lett.* **2016**, *18*, 6, 1386–1389. Copyright (2016) American Chemical Society.

Several papers report cases of “rollover” activation related to phenyl ring rotation instead of heteroaromatic ring rotation. This generalization of the rollover concept is interesting, because it enlarges the field to an ample series of internal ring rotations, even though a “true” rollover process should entail chelation and internal rotation of heteroaromatic rings. However, an interesting example of this enlarged interpretation is the ([RhCp*Cl_2_]_2_ amination of arenes, using pyridine as a directing group. The “rollover” reaction, promoted by water, proceeds with selectivity on a wide range of substrates (both electron-deficient and electron-rich), affording both mono- and di-aminated products **148** and **149** (Figure 82). In this case, rotation of a cyclometalated phenyl ring is a key step of the process, and the pyridine nitrogen is assumed to retain its coordination to the rhodium center [154].

A second case of “rollover” cyclometalation involving a phenyl ring rotation regards the selective bis-cyanation of arylimidazo[1,2-α]pyridines by means of double C–H bond activation, once again with[RhCp*Cl_2_]_2_ as catalyst. The reaction, which proceeds with wide functional group tolerance, enable the synthesis of various cyanated imidazopyridines in high yields; the reaction protocol was also explored toward imidazo containing heterocycles [155].

#### 6.2.2. Palladium

At variance with rhodium, only a few cases of palladium-catalyzed reactions based on a rollover pathway were published in the course of the last years. In these cases, Pd(OAc)_2_ was used as catalyst.

**Figure 83:**
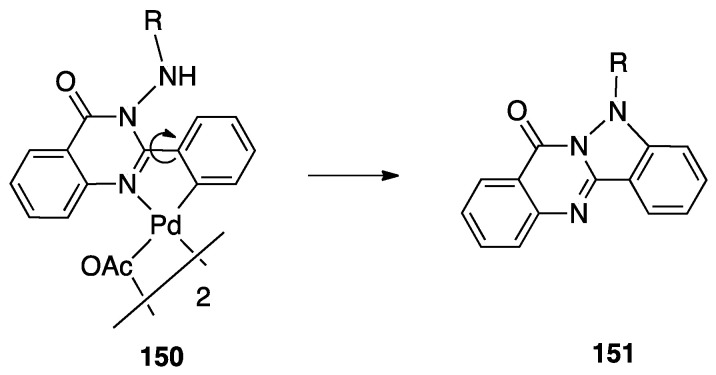
Pseudo-rollover intramolecular oxidative C–H amination of 2-aryl-3-(arylamino)quinazolinones. Adapted with permission from *Org. Lett*. **2014**, *16*, 20, 5418–5421. Copyright (2014) American Chemical Society.

The first palladium-catalyzed process with an internal rotation of a pyridine-like ring regards the intramolecular aerobic oxidative C–H amination of 2-aryl-3-(arylamino)quinazolinones; the reaction afforded a series of fluorescent indazolo[3,2-b]quinazolinones derivatives, **151**, in moderate to excellent yields (Figure 83). Mechanistic evidences suggested the cyclometalated palladium(II) dimer **150** as the key intermediate, which, after cyclometalation, underwent an internal rotation of the quinazolinone ring. The reaction should be classified as “pseudo-rollover” due to the absence of C–H bond activation in the heteroaromatic ring. It is worth to note that this approach to indazolo[3,2-b]quinazolinones has a “green” significance: in the course of the process, reaction with O_2_ as oxidant generates water as the only byproduct. Furthermore, the reaction products have potential utility as a new class of blue fluorophores for fluorescent materials [156].

**Figure 84:**
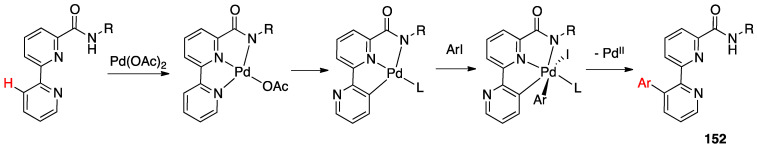
Proposed reaction mechanism (Pd(OAc)2, Cs2CO3, mesitylene, 160 °C, 24 h). Adapted with permission from *Org. Lett*. **2018**, *20*, 4732–4735. Copyright (2018) American Chemical Society.

Cheng and coworkers worked on Pd(II) catalyzed functionalization of 2,2′-bipyridine-6-carboxamides by means of rollover cyclometalation pathways. The researchers showed that Pd(II) acetate catalyzes the arylation of 2,2′-bipyridine-6-carboxamides with aryl iodides in the presence of a base. Despite the presence in the substrate of several sites available for rollover C–H activation, the reaction is regioselective, activating only the external pyridine ring to give **152** as the final products [157]. The authors, on the basis of their experimental results, propose a mechanism, reported in Figure 84, consisting in coordination of the tridentate N^N^N bipy ligand (pre-rollover complex, isolated and characterized), rollover cyclometalation only on the external pyridine ring, oxidative addition of aryl iodide to form a Pd(IV) complex, reductive elimination of the arylated product. Due the great array of substrates studied, the reaction has a broad applicability.

**Figure 85:**
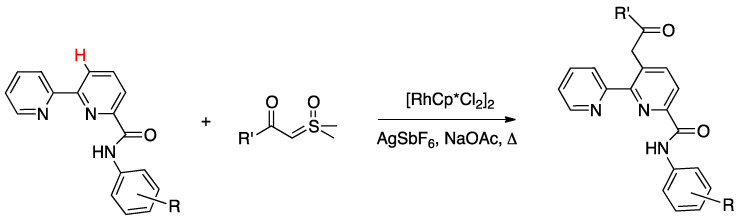
Exclusive regioselective C–H bond functionalization of 2,2′-bipyridine-6-carboxamides. Adapted with permission from *Org. Lett.* **2019**, *21*, 6366–6369. Copyright (2019) American Chemical Society.

It is worth to note that in a successive paper [135], the functionalization of the same class of ligands, catalyzed by [RhCp*Cl_2_]_2_, acted regioselectively on the *internal* C(3)-H bond (see Figure 85). This constitutes a very interesting case of two different rollover regioselective functionalizations, where Pd(II) and Rh(III) catalysts activate in a selective way two different pyridine rings by means of distinctive rollover pathways.

**Figure 86:**
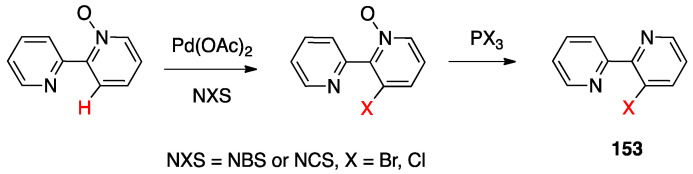
Pd(II) rollover-catalyzed synthesis of 3-halogenated bipyridines. Adapted with permission from *J. Org. Chem*. **2017**, *82*, 11, 5616–5635. Copyright (2017) American Chemical Society.

As previously shown, an easy rollover metalation occurs with *N*-oxide-2,2′-bipyridine (see Section 4.1.2). Taking advantage of this, Tzschucke and coworkers studied the rollover C(3)–H halogenation of bipyridine *N*-oxides by reaction with *N*-chloro- or *N*-bromo-succinimide (NCS or NBS) using Pd(OAc)2 as catalytic precursor. C(3)-H pyridine functionalization gave 3-chloro- or 3-bromobipyridine *N*-oxides with high yields [158]. The authors found that the reaction is highly sensitive to steric hindrance in positions 4 and 6′. In the case of a 6′-substituted ligand coordination to palladium being hindered, and 3′-halogenation was observed. Deoxygenation with PCl_3_ or PBr_3_ allowed the obtainment of 3-halogenated bipyridines **153** (Figure 86).

#### 6.2.3. Rhenium

**Figure 87:**
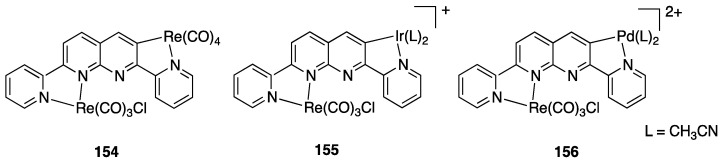
Homo and heterodimetallic Re complexes.

The ligand 2,7-dipyridinyl-1,8-naphthyridine can coordinate in different ways. This ligand has two external pyridine rings, making it able to follow rollover pathways and, consequently, to construct dinuclear complexes. Under certain conditions, a rhenium(I) mononuclear complex was obtained and used for the synthesis of dinuclear complexes. The first coordination involves a simple N^N chelation of the ligand, whereas the second metal promotes a rollover cyclometalation. In this way, homo Re-Re, **154**, and heterodimetallic Re-Ir, Re-Pd, **155** and **156**, complexes were prepared and characterized (Figure 87).

**Figure 88:**
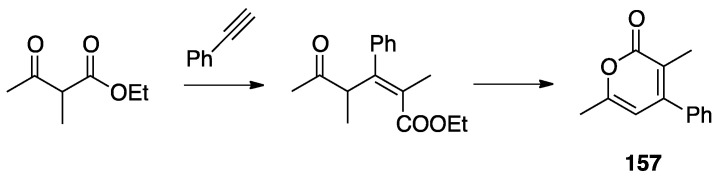
Insertion of terminal acetylenes into b-keto esters catalyzed by complex **154**.

Due to the capacity of rhenium carbonyl complex to catalyze the insertion of terminal acetylenes into b-keto esters, it was investigated the catalytic activities of these heterobimetallic complexes on the same reaction, without any use of organic solvents. Among the three dinuclear rollover complexes, only the dimetallic Re-Re complex **154** showed to be active in the insertion of acetylenes into β-keto esters, under photoirradiation at 350 nm, affording the corresponding 5-oxo-2-hexenoates **157** with high yields (Figure 88) [159]. The heterodimetallic complexes showed, at variance, scarce catalytic activity.

## 7. Applications of Rollover Complexes

Even though this review is dedicated to aspects regarding C–H bond activation and functionalization, it may be of interest to furnish a brief survey of some potential applications of rollover cyclometalated complexes. This will highlight the importance assumed by the reaction and its outcomes. In the course of the last years, several potential applications were found. Synthetic and catalytic applications have been reported in Section 5 and Section 6, other fields involve antitumor compounds, chemosensors, and OLED emitters.

Pt(II) rollover complexes with 2,2′-bipyridines have been investigated for their antitumor properties. In 2017, Shahsavari and coworkers evaluated the biological activity of a series of Pt(II) rollover complexes with 2,2′-bipyridine *N*-oxide and ancillary phosphorus ligands (Section 4.1.2) against a panel of standard cancer cell lines. Two of the complexes showed a potent cytotoxic activity [53]. One year later, the same group extended the study to a series of cycloplatinated complexes (comprising rollover species) bearing 1,10-bis(diphenylphosphino)ferrocene (dppf) ligand. The authors reported the biological evaluation of the species as well as molecular docking studies. The dppf-containing rollover complexes exhibited strong interactions with DNA as well as high cytotoxicity and apoptosis-inducing activities to human cancer cell lines [160]. In the same year, an in vitro study demonstrated that one of the above complexes has better cytotoxicity effect against breast cancer cell lines than *cis*-platin [161].

Again, in the same year, Babak, Hartinger, and coworkers presented an important work on the anticancer activity of a series of mono- and dinuclear Pt(II) rollover complexes with the simple 2,2-bipyridine ligand. The complexes demonstrated to be potent antitumor agents both in vitro and in vivo. Substitution of the neutral and anionic co-ligands on the Pt(k2NC-2,2′-bipyridyl) backbone allowed to establish structure–activity relationships. The authors also showed that the properties of the complexes can be tuned in order to “target different cancer pathways” and “overcome the side effects associated with platinum compounds in cancer chemotherapy” [162].

As previously commented, in addition to Pt(II) derivatives, also Pt(IV) rollover complexes have shown high cytotoxicity towards cancer cells [46].

**Figure 89:**
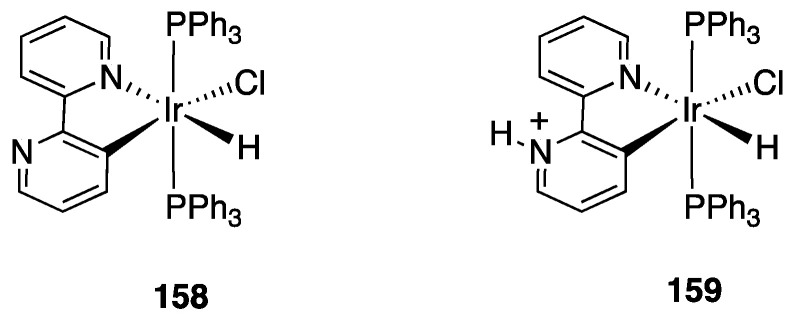
Ir(III) complexes as multi-responsive luminescent materials.

An interesting potential application of Ir(III) rollover complexes was presented in 2015 by Laskar and coworkers. The complex [Ir(PPh3)2(N^C)(Cl)(H], **158** (Figure 89), showed properties that enable it to be employed as a multi-responsive luminescent material [163].

The protonated form of the complex, **159**, has been proven to be useful as a probe for solvents able to form H-bonds. The reversible protonation of complex **158** results in a dramatic change in emission color from bluish-green to yellowish-orange and vice versa after successive protonation and deprotonation reactions.

This behavior allows the rollover complex **158** to act in several ways, as a phosphorescent acid sensor both in solution and in the solid state, and as a chemosensor for detecting acidic and basic organic vapors. In addition, the protonated form, [Ir(bipy-*H*)H^+^], which is generated after protonation of [Ir(bipy-*H*)], can be used as a “solvatochromic probe for oxygen containing solvents”.

Further potential applications arise from the reversible protonation of the uncoordinated nitrogen in rollover helicenes **43** and **44**, which allowed these organometallic compounds to act as “multifunctional switchable systems” [75].

Finally, the potential for the construction of emissive materials was also shown by the double rollover Pt(II) complex **39**, described above [61], with potential applications as OLEDs.

## 8. Conclusions and Perspectives

Even after several years from its recognition as a distinct process, rollover cyclometalation can be still considered a relatively unexplored topic. Investigations in this field still constitute a small argument in chapters dedicated to cyclometalation, although their weight is constantly increasing.

In our opinion, the field has only been marginally explored; as a matter of fact, only a few ligands have been investigated, among the plethora of heterocyclic ligands potentially available.

In general, every bi- or polydentate ligand having two or more heterocyclic rings, sufficiently flexible to allow rotation of the rings and with C–H bonds “on the other side” (usually in symmetric position to one of the donor atoms), is potentially able to follow a rollover metalation. This allows a vast number of ligands to be studied, expanding the area to five- or six-membered heterocyclic rings, metallacycles of different size (e.g., five-, six-, or seven-membered rings) and different donors (N, S, P, C(sp^2^), C(sp^3^), normal or abnormal heterocyclic carbenes, etc.).

As for all cyclometalated complexes, properties may be fine-tuned by modifications of the stereoelectronic properties of substituents on the heterocyclic rings, and by an adequate choice of coligands, oxidation state, and geometry.

Whereas the chemistry of classical cyclometalated complexes has become a mature field of research, the same is far to be true for the rollover behavior.

Mechanistic concepts have been only partly cleared up; whereas the C–H bond activation step is expected to follow related mechanisms in classical and non-classical cases, the factors which drive the conversion of stable chelated complexes into the corresponding rollover complexes is still an obscure matter: only for a few metals, methods for this conversion have been found. A great advancement of the field will be achieved by understanding this crucial step, which could allow the activation of a great number of chelated complexes and metals.

On the whole rollover, cyclometalation constitutes a highly resourceful reaction with applications related to that of classical cases, enriched by the presence of the uncoordinated donor atom. This presence, which enables rollover ligands to be defined as “ligands with multiple personalities”, furnishes enhanced possibilities in biomedicine, for example, due to hydrogen-bonding interactions, in catalysis, due the reversibility of the rollover process (retro-rollover and hydrogen transfer reactions), in material chemistry (e.g., synthesis of homo and hetero-bimetallic systems with metals connected by a highly delocalized ligands), etc.

Future developments in these fields are difficult to foresee, due to ample variability in metal, ligands, and substituents available, added to persisting obscurities in mechanistic aspects (both on the rollover process and on the behavior of rollover complexes). However, we can expect developments in catalyzed C-C coupling processes, hydrogen transfer reactions, photocatalysis, promoted by metals not yet investigated (e.g., Au, Pt, early transition metals, etc.). Advancements in biomedicine (e.g., antitumor and antimicrobial compounds) and material chemistry (sensors, switches, photosensitive devices, etc.) will be expected as well.

However, it can be predicted that further progresses in the area will provide new unforeseen applications and developments contributing to enrich this promising field of organometallic chemistry.
